# Therapeutic Effect of *Jinzhen* Oral Liquid for Hand Foot and Mouth Disease: A Randomized, Multi-Center, Double-Blind, Placebo-Controlled Trial

**DOI:** 10.1371/journal.pone.0094466

**Published:** 2014-04-10

**Authors:** Jun Liu, Guo-Liang Zhang, Gui-Qin Huang, Li Li, Chun-Ping Li, Mei Wang, Xiao-Yan Liang, Di Xie, Chang-Ming Yang, Yan Li, Xiu-Rong Sun, Hong-Sen Zhang, Bai-Song Wan, Wei-Hua Zhang, Hao Yu, Ru-Yang Zhang, Ya-Nan Yu, Zhong Wang, Yong-Yan Wang

**Affiliations:** 1 Institute of Basic Research in Clinical Medicine, China Academy of Chinese Medical Sciences, Beijing, China; 2 Department of Infectious Disease, First Affiliated Hospital of Anhui University of Traditional Chinese Medicine, Hefei, Anhui, China; 3 Department of Infectious Disease, Infectious Disease Hospital of Cangzhou City, Cangzhou, Hebei, China; 4 Department of Infectious Disease, The Fifth People's Hospital of Guiyang City, Guiyang, Guizhou, China; 5 Department of Infectious Disease, Infectious Disease Hospital of Tangshan City, Tangshan, Hebei, China; 6 Department of Epidemiology and Biostatistics, Nanjing Medical University, Nanjing, Jiangsu, China; The George Washington University Medical Center, United States of America

## Abstract

**Background:**

No specific antiviral agent against hand foot and mouth disease (HFMD) is available for clinical practice today.

**Objective:**

To evaluate the efficacy and safety of *Jinzhen* oral solution in treating uncomplicated HFMD.

**Methods:**

In this randomized, double-blind, placebo-controlled trial, 399 children aged 1 to 7 years with laboratory confirmed HFMD were randomized to receive *Jinzhen* oral liquid or placebo 3 times daily for 7 days with a 3-day follow-up. The primary outcomes were time to the first disappearance of oral ulcers and vesicles on hand or foot and time to the first normalization of temperature (fever clearance).

**Results:**

There were 199 children enrolling into the *Jinzhen* group including 79 with fever and 200 into the placebo group including 93 with fever. *Jinzhen* reduced the time to the first disappearance of oral ulcers and vesicles on hand or foot to 4.9 days (95% CI, 4.6 to 5.2 days), compared with 5.7 days (95% CI, 5.4 to 6.0 days) in the placebo group (P = 0.0036). The median time of fever clearance was shorter in the 79 children who received *Jinzhen* (43.41 hrs, 95% CI, 37.05 to 49.76) than that in the 93 children who received placebo (54.92 hrs, 95% CI, 48.16 to 61.68) (P = 0.0161). Moreover, *Jinzhen* reduced the risk of symptoms by 28.5% compared with placebo (HR, 0.7150, 95% CI, 0.5719 to 0.8940, P = 0.0032). More importantly, treatment failure rate was significantly lower in the *Jinzhen* group (8.04%) compared with that in the placebo group (15.00%) (P = 0.0434). The incidence of serious adverse events did not differ significantly between the two groups (9 in *Jinzhen* group vs. 18 in placebo, P = 0.075).

**Conclusions:**

Children with HFMD may benefit from *Jinzhen* oral liquid treatment as compared with placebo.

**Trial Registration:**

Chinese Clinical Trial Registry (http://www.chictr.org/en/) ChiCTR-TRC-10000937

## Introduction

In the past three decades, several severe outbreaks of hand, foot and mouth disease (HFMD) took place throughout the world, especially in the Asia-Pacific region [1]. The epidemic of HFMD broke out in Taiwan in 1998, leading to 129,106 cases of HFMD, 405 severe cases with complications [2], and even 78 deaths [3]. And then it was followed by outbreaks in other parts of the Asia-Pacific region, including Singapore, Australia and Japan. [4], [5] HFMD epidemics have been a serious public health concern in China since 2007. The recent epidemic in 2010, which was manily caused by coxsackievirus A16 (CVA16) and enterovirus 71 (EV71), involved 1,774,669 cases of HFMD, resulting in 905 deaths [6]. The viruses are members of the Picornaviridae family and can be spread through contact with virus-containing body fluids, respiratory droplets, and feces. No specific antiviral agent or vaccine is now available for HFMD for clinical practice. Although ribavirin and immunoglobulin are commonly used, the efficacy remains uncertain [7]. Therefore, good personal hygiene, including hand washing and disinfection of surfaces in child care facilities was recommended as the most effective approach to reduce the transmission rate of HFMD [8].

During several recent epidemics of HFMD in China, some of the herbs or herbal preparations have shown therapeutic efficacy against the disease, including ameliorating the symptoms and shortening the course of HFMD [9]. *Jinzhen* oral liquid, a kind of Chinese patent medicine extracted from the herbs including baikal skullcap root, rhubarb, gypsum, etc. (Table S1), approved by China Food and Drug Administration (CFDA), has been used to treat fever induced by some childhood viral-infectious diseases for decades and has been recorded into “Pharmacopoeia of the People's Republic of China in 2010” [10]. *Jinzhen* oral liquid could inhibit the replication of EV71 and Cox A16 in vitro [11]. The major components in this oral liquid have been demonstrated to have some active pharmacological functions for viral-infectious diseases, such as antipyretic activities (baikal skullcap root [12]), immunoregulatory and anti-inflammatory effects (rhubarb [13], baikal skullcap root [14], [15]), antiviral properties (baikal skullcap root [16]), etc. Besides, a pilot clinical study conducted in 42 children with HFMD in China suggested that *Jinzhen* oral liquid combined with ribavirin could both reduce the duration of fever and the time to the first disappearance of oral ulcers and vesicles on hand or foot, compared with only use of ribavirin [17]. However, there was insufficient evidence to support its widespread clinical use in the treatment of HFMD.

Therefore, we conducted a double-blind, randomized, placebo-controlled trial to assess the antipyretic and anti-inflammatory benefits of *Jinzhen* compared with placebo. We hypothesized that patients in the *Jinzhen* group would have greater reductions in time to the first disappearance of oral ulcers and vesicles on hand or foot and time of fever clearance.

## Methods

### Trial Design and Ethics Statement

This is a randomized, double-blind, parallel, placebo-controlled trial with an allocation ratio of 1∶1. The study protocol was approved by the institutional review board of the Institute of Basic Research in Clinical Medicine (IRB-IBRCM-2010-NO 3) in China Academy of Chinese Medical Sciences. After obtaining the approval of the Institutional Review Board (IRB) on May 30th 2010, we started to prepare the trial registration. However, since HFMD is a seasonal infectious disease which highly took place in April to July, the first patient was recruited on June 30th, 2010 in the Fifth People's Hospital of Guiyang City, which is located in West of China and lack of awareness of trial registration. After getting the news of recruitment, we accelerated our process of trial registration and finally completed the trial registration on July 4th, when the first patient had not completed the treatment. The authors confirmed that all ongoing and related trials for this drug were registered at the Chinese Clinical Trial Registry (ChiCTR-TRC-10000937). The protocol for this trial and supporting CONSORT checklist are available as supporting information; see Checklist S1 and Protocol S1.

### Participants

Participants in hospital were recruited from June 30th 2010 through November 2010 in 3 centers across China: The Fifth People's Hospital of Guiyang City, Infectious Disease Hospital of Cangzhou City and Infectious Disease Hospital of Tangshan City. Samples from the throat or stool were sent to the laboratory of Chinese Centre for Disease Control and Prevention to determine that which non-polio enteroviruses caused the illness by the real time polymerase chain-reaction (RT-PCR) test.

Patients aged 1 and 7 years who have been clinically diagnosed as uncomplicated HFMD if they had at least one of the following features accompanied or not accompanied by fever: maculopapular of vesicular rash on the palms and/or soles and vesicles or ulcers in the mouth according to the “Diagnosis and treatment guidelines of HFMD (2010 edition)” issued by Ministry of Health of China [18] were recruited into the trial, with a history of vesicles or fever with a maximum duration of 48 hrs and a temperature no more than 39°C. We excluded those children who were complicated with congenital heart disease, chronic hepatitis, nephritis or hematological diseases; those who were prone to allergies or with known allergy to the study drug; those or their guardians with psychiatric disorders; those with chronic diarrhea; and those who were participating other clinical trials. All parents or guardians of the eligible children provided written informed consent.

### Interventions

Inpatient children were randomly assigned to receive 1 mL of *Jinzhen* or its matched placebo (both produced by Kanion Pharmaceutical Co. Ltd) per kilogram body weight every day for 7 days, followed by a 3-day follow-up. The amount of the every-day drug would be taken orally for three times a day using a calibrated syringe. All liquid were sugar-free. Since it's reported that the symptoms in some cases of HFMD disappeared after 48 hrs [19], we asked parents to give the drugs three times per day in the first 2 days. And from Day 3 to Day 7, if their child's symptoms persisted, parents should give the drugs three times per day right along until all symptoms were disappeared. At the end of Day 7, we terminated the drug delivery.

The ibuprofen suspension (Motrin, Johnson & Johnson) was also used for treating children whose temperature exceeded 38.5°C. Usually, it was administrated once every 4–6 hrs if the temperature did not decrease under 38.5°C, but no more than 4 times a day.

### Outcomes

The primary outcomes were time to the first disappearance of oral ulcers and vesicles on hand or foot within 10 days (including 3-day follow up), and time to the first normalization of temperature (fever clearance). The starting points of these two time were both being calculated from the moment of symptom onset. The axillary temperature was measured using mercury-thermometers for 5 mins each time and recorded in the temperature card by the children's parents with the help of investigators every 4 hrs each day. If the temperature was tested normal (<37°C) more than 3 times, recording of temperature could be terminated. The investigators also recorded the status of the oral ulcers and vesicles on hand or foot daily. When all of the oral ulcers and vesicles on hand or foot disappeared, defined as “no new ones appearing” determined by the experienced clinicians, the investigator recorded the date as the time for the first disappearance of oral ulcers and vesicles.

With the help of investigators, the parents also completed symptom diaries by assessing if their child had the following symptoms or not: vomiting, salivation, expectoration, diarrhea, nasal discharge, poor appetite, hypersomnia, cough, constipation, and dysphoria. And then investigators recorded the proportion of the children who were free of these symptoms every day as a secondary outcome. If used, the frequency and dose of ibuprofen were also recorded. Treatment failure rate was another important outcome, defined as the occurrence of any of the following conditions [20]: (1) severe disorders (such as encephalitis); or (2) white blood cell (WBC) counts >10,000/mm^3^.

Throughout the 7-day intervention period, all the patients should be kept in the hospital unless they were healed, and the investigators monitored all adverse events, using a standard adverse-event case report form to collect information about the adverse event if occurred. And the investigators would also assess if the adverse event was related to the drug or not. The form contained a description of all unanticipated events and undesirable experiences, particularly exacerbations of HFMD symptoms.

### Sample size

We assumed an exponential distribution for the time-to-event outcomes of the two groups in sample size calculation using log-rank test. As concerning the co-primary outcomes, the type-1 error (α) should be adjusted to 0.025 by Bonferroni method. On the assumption of a reduction in fever clearance of 16 hrs in *Jinzhen* group compared with the average time of fever clearance in placebo group (64 hrs) [7], [19] and a reduction of a reduction in time to the first disappearance of oral ulcers and vesicles of 1.2 days in *Jinzhen* group compared with that in placebo group (5.46 days) [7], a two-tailed α risk of 2.5%, a loss proportion of 20%, 460 evaluable people were needed (230 per group) to ensure a statistical power of 80%.

### Randomization allocation and blinding

Eligible people were randomly assigned as 1∶1 ratio to each group, allocated a four-digit randomization number through a central interactive web response system. The randomization list, generated by an independent clinical research organization via PROC PLAN process of SAS 9.1.3, linked sequential numbers to the treatment allocated at random. The out-put randomization program, kept in seal by this independent organization, included serial-number of the subjects, random number of the subjects, group number of the subjects and the drug number.

The children's parents, investigators (including physicians, nurses and other clinical personnels involved in the daily clinical care of the patients), and statisticians were all blinded to treatment allocation by using identically matched placebos, which was identical in appearance, color, taste and smell with *Jinzhen* oral liquid (the overall similarity = 93.225%) (File S1). The placebo was made from edible flavor, edible food colors, cane sugar and water (Figure B in File S1). All of the drugs and placebos were produced by the Kanion Pharmaceutical Co. Ltd, and all of the investigators and statisticians were not involved in the preparation of the drug and placebo.

### Statistical analysis

The primary outcome measures were time-to-event variables with survival curves made by the Kaplan-Meier method, and comparisons between the two groups were performed via the log-rank test; hazard ratios (HRs) and 95% confidence intervals (CIs) were calculated through a Cox regression model. We also tested the potential interactions between treatment and covariates, including age, gender, contact history with patients with HFMD, coexisting illnesses, health status, and medication. Outcomes or proportion of patients with adverse events were compared between the two groups by the Fisher's exact test and binomial 95% CIs. All analyses were performed on data from the intention-to-treat population. All reported *P* values were two-sided, and all the *P* values were considered to be statistically significant if less than 0.05.

## Results

### Patients' disposition and the general characteristics

**Figure 1:**
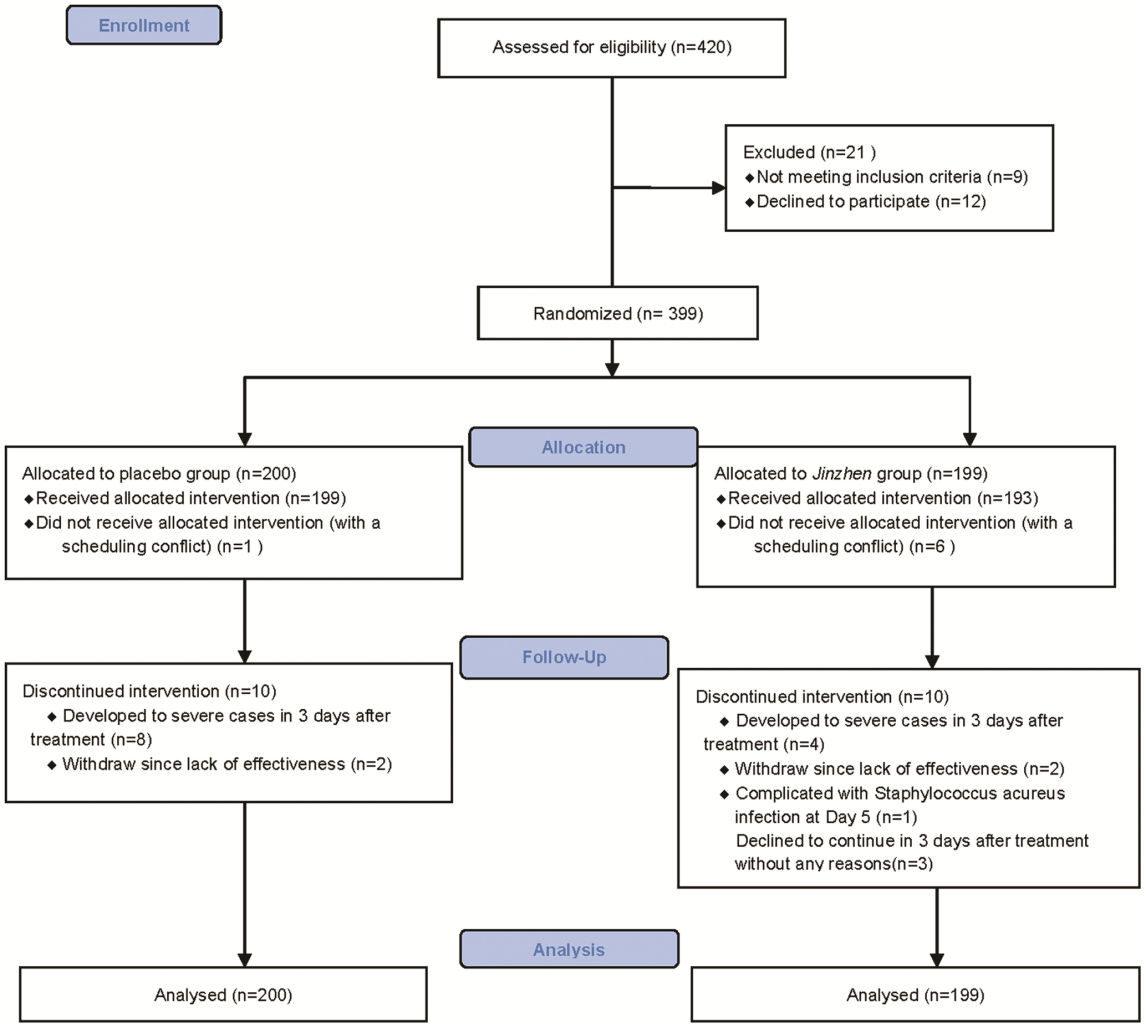
The Flow Diagram of the Trial.

We originally planned to screen 460 in-hospital patients in 3 hospitals from June 30th 2010 to November 2010. However, since HFMD is a seasonal contagious disease which often occurs in summer from April to October in China, we could not enroll any more patients after November. Therefore, we ended the trial in November 2010, and a total of 420 patients were recruited in our study. Recruitment flow, including participant retention throughout the study, is shown in Figure 1.

There were no statistically significant differences between the two groups on any baseline measure (Table 1). The mean days of drug use in the *Jinzhen* group (4.6±1.2 d) were fewer than that in the placebo group (4.9±1.3 d) (*P* = 0.0215), while the mean days of drug use in the subgroup of children with fever were similar between the two groups (4.5±1.3 d in *Jinzhen* group vs. 4.7±1.5 d in placebo group, *P* = 0.2155).

**Table 1:** Baseline characteristics of study participants.

	All study participants		Participants with fever*	
Characteristics	*Jinzhen* (n = 199)	Placebo (n = 200)	*Jinzhen* (n = 79)	Placebo (n = 93)
Male sex, No. (%)	127(63.82)	113(56.50)	57(72.15)	55(59.14)
Age, mean ±SD (years)	2.4±1.2	2.4±1.2	2.5±1.3	2.4±1.4
Weight, mean ±SD (kg)				
Male	12.70±2.35	12.98±3.09	12.78±2.54	13.28±3.44
Female	12.56±3.07	12.44±3.33	13.17±3.31	12.03±3.17
Ethnic groups, No. (%)				
Han	193(96.98)	187(93.50)	78(98.73)	88(94.62)
Others	6(3.02)	13(6.50)	1(1.27)	5(5.38)
Source of case, No. (%)				
Children kept at home	174(87.44)	167(83.50)	67(84.81)	81(87.10)
In kindergarten	21(10.55)	30(15.00)	10(12.66)	10(10.75)
Pupils	3(1.51)	2(1.00)	2(2.53)	2(2.15)
Others^a^	1(0.50)	1(0.50)	0(0.00)	0(0.00)
Temperature, mean ±SD (°C)	37.51±0.83	37.61±0.80	37.89±0.71	38.08±0.56
Contact history with HFMD within 1 week before onset, No. (%)				
Yes	49(24.62)	54(27.00)	21(26.58)	23(24.73)
No	150(75.38)	146(73.00)	58(73.42)	70(75.27)
Symptoms at onset —No. (%)				
Fever	16(8.04)	17(8.50)	4(5.06)	7(7.53)
Vesicles	54(27.14)	57(28.50)	7(8.86)	7(7.53)
Fever and vesicles	125(62.81)	124(62.00)	67(84.81)	79(84.95)
Others	4(2.01)	2(1.00)	1(1.27)	0(0.00)
Medication use before intervention, No. (%)				
Yes	101(51.01)	115(57.50)	45(56.96)	58(62.37)
No	97(48.99)	85(42.50)	34(43.04)	35(37.63)
Previous history with HFMD, No. (%)				
Yes	2(1.01)	0(0.00)	0(0.00)	0(0.00)
No	197(98.99)	200(100.00)	79(100.00)	93(100.00)
Concomitant symptoms, No. (%)				
Yes	165(82.91)	167(83.50)	73(92.41)	85(91.40)
No	34(17.09)	33(16.50)	6(7.59)	8(8.60)
Enterovirus71, No. (total)^b^	27(114)	31(100)	14(45)	17(46)
CoxsackievirusA16 —No.(total)^c^	13(112)	12(100)	4(45)	2(46)
Other enteroviruses —No. (total)^d^	30(55)	22(44)	11(19)	4(17)
WBC counts>10,000/mm^3^—No. (total)^e^	95(198)	99(199)	38(79)	48(92)

*Participants with fever were defined as those children whose last temperature before intervention was greater than or equal to 37.5°C.

a “Others” referred to those who could not been recorded “the source of case” clearly in CRF.

b “No.” referred to the number of patients with Enterovirus71 virus-positive, while “(total)” referred to the total number of patients who underwent the detection of Enterovirus71 virus. In the first column, for example, “27(114)” indicated that 114 patients in the *Jinzhen* group underwent the detection of Enterovirus71 virus and 27 of them were identified as Enterovirus71 virus-positive.

c “No.” referred to the number of patients with Coxsackievirus A16 virus-positive, while “(total)” referred to the total number of patients who underwent the detection of CoxsackievirusA16. In the first column, for example, “13(112)” indicated that 112 patients in the *Jinzhen* group underwent the detection of CoxsackievirusA16 virus and 13 of them were identified as CoxsackievirusA16 virus-positive.

d “No.” referred to the number of patients with other enteroviruses -positive (such as CoxsackievirusA10, CoxsackievirusA6, etc.), while “(total)” referred to the total number of patients who underwent the detection of other enteroviruses. In the first column, for example, “30(55)” indicated that 55 patients in the Jinzhen group underwent the detection of other viruses and 30 of them were identified as other enteroviruses-positive.

e “No.” referred to the number of patients whose WBC counts>10,000/mm^3^, while “(total)” referred to the total number of patients who underwent blood routine test. Data of all participants were missing for one child from each of the two groups, while data of patients with fever was missing for one child in the placebo group.

### Primary outcomes

**Figure 2:**
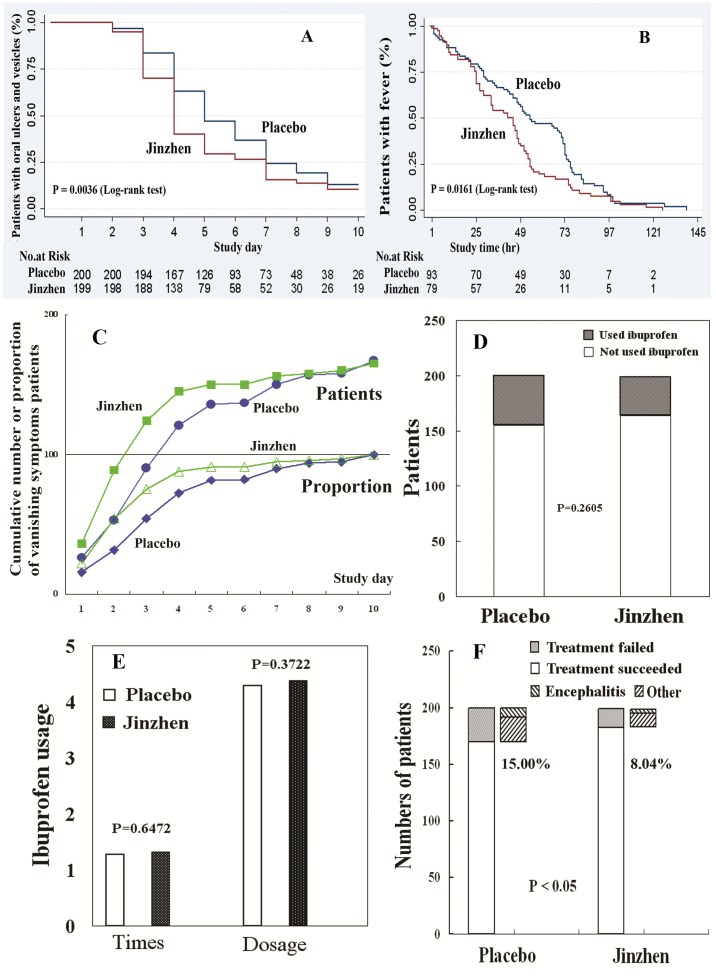
Outcome measures analysis between *Jinzhen* and placebo groups. Panel A: Kaplan-Meier curves for the time to the first disappearance of oral ulcers and vesicles; Panel B: Kaplan-Meier curves for the time of fever clearance; Panel C: The risk of HFMD symptoms both in the number and proportion of patients. Panel D: The combined use of ibuprofen. Panel E: The frequency or dose of ibuprofen used. Panel F: The treatment failure rate.

Compared with placebo, *Jinzhen* reduced the time of oral ulcers and vesicles on hand or foot by 24% within 10 days (including a 3-day follow-up). The median time to the first disappearance of oral ulcers and vesicles on hand or foot was shortened to 4.9 days (95% CI, 4.6 to 5.2 days) in the *Jinzhen* group, compared with 5.7 days (95% CI, 5.4 to 6.0 days) in placebo group (HR: 0.76, 95% CI: 0.62 to 0.94, *P* = 0.0036) (Figure 2A). *Jinzhen* also reduced the risk of fever by 31.87% when compared with placebo. The median time of fever clearance was marginally shorter in the *Jinzhen* group (43.41 hrs, 95% CI, 37.05 to 49.76) than that in the placebo group (54.92 hrs, 95% CI, 48.16 to 61.68) (HR: 0.6823; 95% CI: 0.4962 to 0.9381, *P* = 0.0161) (Figure 2B).

**Figure 3:**
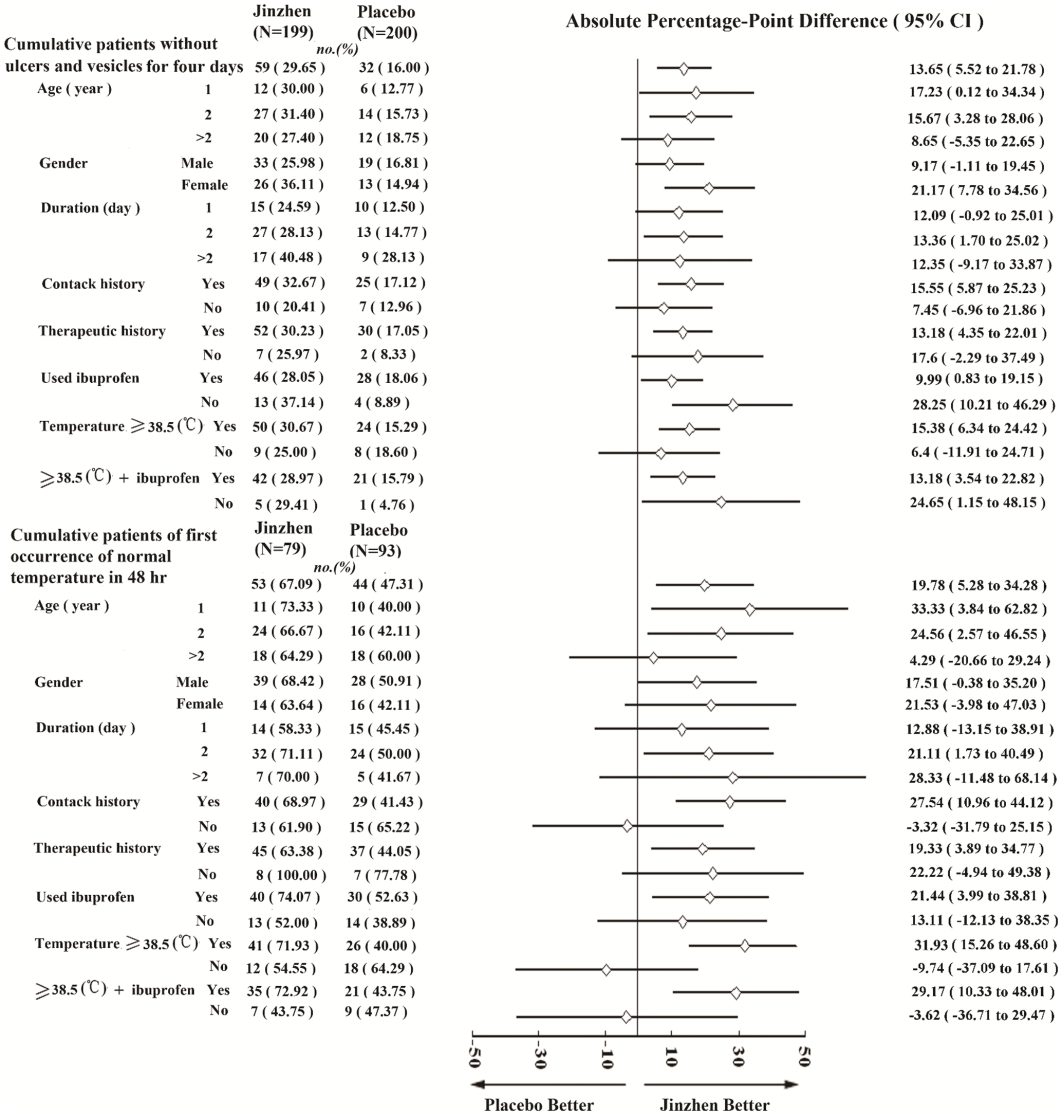
Subgroup analyses of absolute risk differences between the *Jinzhen* and placebo groups in patients. In the subgroup analysis of the primary outcome measures, a patient was counted only once.

Subgroup analysis of patients without oral ulcers and vesicles on hand or foot at Day 4 indicated that *Jinzhen* was superior to placebo in children under 2 years of age, female gender, having a contact and medication history, with a temperature above 38.5°C, or who used ibuprofen. Fever clearance was achieved at 48 hrs in 53 children in the *Jinzhen* group (53/79, 67.09%) and 44 children in the placebo group (44/93, 47.31%), indicating the superiority of *Jinzhen* over placebo (*P* = 0.0091). Besides, the same results were also obtained in children who had a contact history, with a temperature above 38.5°C, or who used ibuprofen. (Figure 3)

Compared with placebo, *Jinzhen* could reduce the risk of oral ulcers and vesicles on hand or foot in 123 virus-positive children (64 in *Jinzhen* group vs. 59 in placebo) by 42.8% (HR,0.572, 95% CI, 0.395 to 0.827, *P* = 0.0030). In 91 virus-negative cases (50 in *Jinzhen* group vs. 41 in placebo), however, no difference was noted between the two groups in shortening the duration of oral ulcers and vesicles on hand or foot (HR, 0.810, 95% CI, 0.526 to1.247, *P* = 0.3377). Also, no differences were identified between the two groups in term of shortening the duration of fever in 49 virus-positive cases (27 in *Jinzhen* group vs. 22 in placebo) (HR, 0.626, 95% CI, 0.338 to 1.159, *P* = 0.1360) or in 42 virus-negative cases (18 in *Jinzhen* group vs. 24 in placebo) (HR, 0.774, 95%CI, 0.385 to 1.555, *P* = 0.4718).

### Secondary outcomes

The number and the proportion of patients whose symptoms disappeared were significantly different between the two groups (*P* = 0.0021). *Jinzhen* reduced the risk of symptoms by 28.5% while compared to the placebo (HR, 0.7150, 95% CI, 0.5719 to 0.8940, *P* = 0.0032) (Figure 2C). The combined use of ibuprofen was not significantly different between the two groups (22.5% in placebo vs. 17.59% in *Jinzhen* group, *P* = 0.2605) (Figure 2D).In addition, the frequency and dose of ibuprofen were also similar between the two groups (Frequency: 1.27±0.45 in placebo vs. 1.31±0.47 in *Jinzhen* group, *P* = 0.6472; Dose: 4.29±0.69 in placebo vs. 4.37±1.26 in *Jinzhen* group, *P* = 0.3722) (Figure 2E). At the end of the intervention period, treatment failure rate was significantly lower in the *Jinzhen* group (16/199, 8.04%) compared to the placebo group (30/200, 15.00%) (HR, 0.5360, 95% CI, 0.3019 to 0.9517, *P* = 0.0434) (Figure 2F).

### Safety

The rates on the loss of follow-up were similar between the two groups (5.03% in *Jinzhen* group vs. 5.00% in placebo group), indicating similar overall tolerability of the study treatments. No vomiting event was reported in the trial. Hematological and biochemical abnormalities were mild and considered not related to the study drugs. The incidence of serious adverse events (SAEs) did not differ significantly between the two groups (9 in *Jinzhen* group vs. 18 in placebo, *P* = 0.075) (Table 2).

**Table 2:** Serious adverse events in the safety population.

	*Jinzhen* (n = 199)	Placebo(n = 200)
**Any Class**	9 (4.52%)	18 (9%)
**Respiratory system disorders**	1 (0.50%)	1 (0.5%)
Bronchopneumonia	1 (0.50%)	1 (0.5%)
**Nervous system disorders**	4 (2.01%)	8 (4%)
Encephalitis	4 (2.01%)	8 (4%)
**Gastrointestinal disorders**	0	1 (0.5%)
Enteritis	0	1 (0.5%)
**Cardiac disorders**	0	1 (0.5%)
Symptomatic sinus tachycardia	0	1 (0.5%)
**Laboratory investigations**	4 (2.01%)	7 (3.5%)
Transiently increased lactate dehydrogenase	4 (2.01%)	5 (2.5%)
Transiently increased alanine transaminase	0	2 (1%)

Data are number (%) of patients with at least one adverse event. The safety population consisted of all participants randomly assigned to treatment groups and treated. MedDRA version 14.0 was used for assessment.

## Discussion

In our study, it showed that *Jinzhen* oral liquid is potentially an effective therapy for patients with HFMD. Compared with the placebo, *Jinzhen* oral liquid could improve the clinical symptoms of HFMD more rapidly, including fever, oral ulcers, vesicles on hand or foot, and other symptoms in multiple organs or systems. The onset time of *Jinzhen* oral liquid for lowering body temperature was probably between 3 hr and 6 hr after administration. In addition, HFMD is a highly contagious disorder that is spread by fecal contamination and contact [21], [22]. Moreover, the intraoral sores often interfere with proper feeding, presenting a risk of dehydration and fluid imbalance, especially in infants. Thus, early control of the vesicle rash could reduce the risk of transmission of HFMD, relieve the corresponding symptoms and improve the quality of daily life for the sufferers. Our results also demonstrated that early treatment with *Jinzhen* oral liquid might be beneficial in reducing the risk of multi-organ exacerbations by lowering the rate of treatment failure. Besides, the tolerability and a low incidence of adverse events of *Jinzhen* oral liquid indicated that this drug was probably a well-tolerated and safe therapy for children with HFMD, which is also easy to be incorporated into the treatment of HFMD. As shown in Table 2, although there were no statistically significant difference of SAEs between the two groups, *Jinzhen* oral liquid seemed to associate with a lower incidence of SAEs. Some of the SAEs, as encephalitis, bronchopneumonia, symptomatic sinus tachycardia, were mostly considered as the severe complications of HFMD, indicating that the lower incidence of these SAEs might be more effective for preventing the mild HFMD to serious, even life-threatening forms of the disease. It might suggest that *Jinzhen* oral liquid had protential advantages for preventing HFMD from exacerbations, which was also justified in relation to the gain in terms of shortened disease duration.

The biologic mechanisms how *Jinzhen* oral liquid affects the clinical course of HFMD remain unknown. On one hand, HFMD is caused by numerous members of the Enterovirus genus e.g. *Cox*A 16 and EV71, which may induce multiple system organ damage [20]. On the other hand, the intervention of *Jinzhen* oral liquid may focus on multiple outcomes based on the spectrum of multi-ingredients to treat HFMD instead of using a single pure chemical to produce a single outcome [23]. Previous reports suggested that baicalin and its aglycone baicalein (Figure A in File S1), one of the major active compounds in *Jinzhen* oral liquid, had a wide range of pharmacological effects, including anti-allergic [24], anti-inflammatory [25], antiviral [26], and antipyretic [12] effects, and also showed strong activities in preventing acute lung injury [27] and neurological dysfunction [28].

Study strengths include (1) its originality: there were only two trials of supportive treatment for HFMD reported in *PubMed*: one was using low-lever laser treating 19 patients with HFMD-induced painful stomatitis [29]; and the other was using milrinone treating 24 children with EV71-induced pulmonary edema [30]. Otherwise, our study pays close attention to the improvement of the whole status of HFMD with a large sample size, considered novel and original. (2) Its internal validity: randomization was concealed, patients, investigators and biostatisticians were blinded to treatment allocation, and confounding factors and bias were minimal. (3) The use of identically matched placebos: We prepared the matched placebos and made a quantitative comparison of the appearance, color, taste and smell between *Jinzhen* oral liquid and its placebo by intelligent sensory technology (the overall similarity = 93.225%) (File S2). The use of identically matched placebo ensured the validity of the double-blind design. (4) We kept the pharmacological effects of *Jinzhen* oral liquid and placebos relatively stable in this trial. The quality control of ingredients in *Jinzhen* oral liquid was performed via high performance liquid chromatography (HPLC) fingerprinting (File S1), and both *Jinzhen* oral liquid and its placebo were manufactured in a same batch strictly accordance with the standards of “good manufacturing practice ”(GMP).

However, we are also aware of four possible weaknesses of the study. Firstly, the facts that the treatment was not delivered under a stratified randomization of viral types and the outcomes did not include the clearance of certain viruses, potentially limited the pertinence of our results. However, we believe that the subject population with an overall poor health status at baseline may be representative of the patients with HFMD in a real-world clinical practice. Thus, it would be prudent to further explore the benefits of *Jinzhen* in treating HFMD caused by the certain virus. Secondly, since we followed participants for only 3 days and the clearance of virus did not occur simultaneously with the disappearance of symptoms, the anti-viral effect of *Jinzhen* oral liquid might require further evaluations with a longer follow-up. Thirdly, to minimize potential confounding factors in this trial, we kept all patients hospitalized, which may have an impact on the generalisability of the study result. In accordance with the published guideline [18], HFMD with mild symptoms could be managed in outpatient clinic. However, according to China's national health reports, HFMD has been the fifth leading cause of death among all contagious diseases since 2010 [6], and if the child with fever, most of the Chinese family would like to leave him/her in-hospital to prevent the disease exacerbating. Indeed, in the future, we'd like to conduct the registry study on *Jinzhen* oral liquid for evaluating its effect on HFMD in real-world clinical practice. Fourthly, in our study, *Jinzhen* Oral liquid was only treated for HFMD with mild symptoms based on its clinical application for decades. However, the severe complications of HFMD, e.g. viral meningitis, encephalitis, pulmonary edema, were the main life-threatening causes. Therefore, for further studies on HFMD, we should pay more attention to the the higher risk cases, e.g. HFMD with severe symptoms of central nervous systems such as high fever, lethargic, drowsy or irritability. We should set main outcome measures or primary endpoints as mortality rates, or survival rates, or occurrence of transition of the disease to serious, even life-threatening forms of the disease. Besides, the evaluation of the antiviral effect also should be considered in the future studies.

## Conclusions

In summary, our study provides evidence that *Jinzhen* oral liquid is beneficial in the treatment of HFMD. This treatment may reduce the risk of treatment failure by improving both the overall condition and clinical symptoms with less serious adverse events. The mechanism of the multiple actions of *Jinzhen* oral liquid needs to be further explored in future clinical pharmacological studies.

## Supporting Information

Checklist S1 **CONSORT 2010 checklist of information to include when reporting a randomised trial.**
(DOC)

File S1 **Fingerprint electropherogram of** ***Jinzhen*** **oral liquid and placebo.**
(DOC)

File S2 **Comparison between** ***Jinzhen*** **oral liquid and Its Placebo by Intelligent Sensory Technology.**
(DOC)

Protocol S1 **The protocol of “** ***Jinzhen*** **oral liquid for Hand Foot and Mouth Disease in Children: a Randomized, Multi-center, Double-blind, Placebo-controlled Trial”.**
(DOC)

Table S1 **The major components in** ***Jinzhen*** **oral liquid.**
(DOC)
