# Supplementary material for: Therapeutic Effect of Jinzhen Oral Liquid for Hand Foot and Mouth Disease: A Randomized, Multi-Center, Double-Blind, Placebo-Controlled Trial
Source: PLoS One. 2014 Apr 10;9(4):e94466. doi: 10.1371/journal.pone.0094466 (PMC3983189; doi:10.1371/journal.pone.0094466)

**File S1. Fingerprint electropherogram of** ***Jinzhen* oral liquid and placebo.**

**Figure A. Fingerprint electropherogram of *Jinzhen* oral liquid.** The main bioactive ingredients of *Jinzhen* oral liquid include Baicalin, Baicalein.


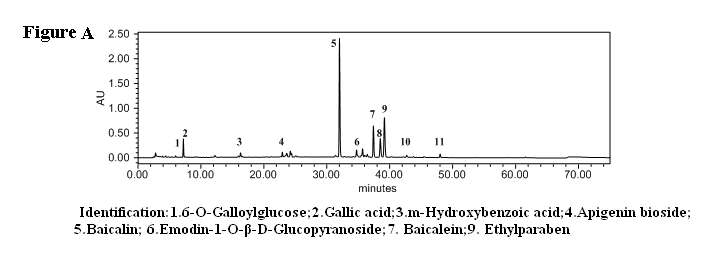


**Figure B. Fingerprint electropherogram of placebo.** The placebo was made from edible flavor, edible food colors, cane sugar and water.


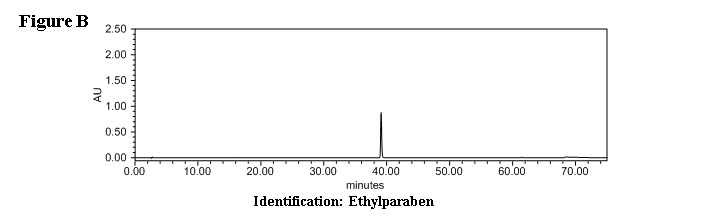

Supplement: File S1 — Fingerprint electropherogram of Jinzhen oral liquid and placebo. (DOC) [file pone.0094466.s002.doc]
