# Supplementary material for: Therapeutic Effect of Jinzhen Oral Liquid for Hand Foot and Mouth Disease: A Randomized, Multi-Center, Double-Blind, Placebo-Controlled Trial
Source: PLoS One. 2014 Apr 10;9(4):e94466. doi: 10.1371/journal.pone.0094466 (PMC3983189; doi:10.1371/journal.pone.0094466)
Supplement: File S2 — Comparison between Jinzhen oral liquid and Its Placebo by Intelligent Sensory Technology. (DOC) [file pone.0094466.s003.doc]

## File S2. Comparison between *Jinzhen* oral liquid and Its Placebo by Intelligent Sensory Technology

The quantitative comparison of the appearance, color, taste, smell between *Jinzhen* oral liquid and its placebo was conducted by intelligent sensory technology including [machine vision](dict://key.0895DFE8DB67F9409DB285590D870EDD/machine vision), electronic nose and electronic tongue technique. The results showed as following:

**1** [**Machine vision**](dict://key.0895DFE8DB67F9409DB285590D870EDD/machine vision) **test**

**1.1 Methods**

The picture of *Jinzhen* oral liquid and its placebo was taken as below by Canon350-D. And the comparison data were obtained using pattern recognition software.

**
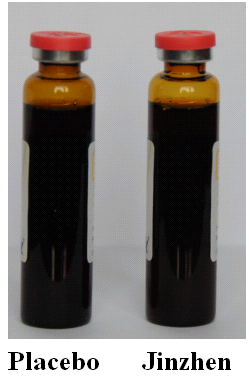
**

**1.2** **The results of** [**machine vision**](dict://key.0895DFE8DB67F9409DB285590D870EDD/machine vision) **test**

**Table 1 The results of** [**machine vision**](dict://key.0895DFE8DB67F9409DB285590D870EDD/machine vision) **test**

|  | *Jinzhen* | Placebo |
| --- | --- | --- |
| R(Mean) | 0.115636 | 0.186034 |
| G(Mean) | 0.571613 | 0.280796 |
| B(Mean) | 0.69306 | 0.238798 |
| R(SD) | 0.134262 | 0.271793 |
| G(SD) | 0.311256 | 0.33989 |
| B(SD) | 0.347439 | 0.305154 |
| R(Entropy) | 1.57202 | 1.542174 |
| G(Entropy) | 2.052138 | 1.572318 |
| B(Entropy) | 1.838894 | 1.599646 |
| similarity | 95.27% | |

The results show that the similarity of the two testing samples in visual appearance is 95.27%.

**2** **Electronic nose test**

**2.1** **Performance of the sensors**

There are 18 sensors in electronic nose. These sensors are all made from metal oxide. The names and performances of these sensors are shown as Table 2.

**Table 2 Performance of the sensors in electronic nose**

| Number | Sensor name | Performance |
| --- | --- | --- |
| 1 | LY/LG | sensitive to the strong oxidizing gas |
| 2 | LY2/G | sensitive to the toxic gas |
| 3 | LY2/AA | sensitive to the [organic compounds](http://dict.cnki.net/dict_result.aspx?searchword=有机化合物&tjType=sentence&style=&t=organic+compounds) |
| 4 | LY2/Gh | sensitive to the toxic gas |
| 5 | LY2/gCT | sensitive to the [inflammable](http://dict.cnki.net/dict_result.aspx?searchword=易燃&tjType=sentence&style=&t=inflammable) gas |
| 6 | LY2/gCT1 | sensitive to the toxic |
| 7 | T30/1 | sensitive to the [organic compounds](http://dict.cnki.net/dict_result.aspx?searchword=有机化合物&tjType=sentence&style=&t=organic+compounds) |
| 8 | P10/1 | sensitive to the [combustible](http://dict.cnki.net/dict_result.aspx?searchword=可燃&tjType=sentence&style=&t=combustible) gas |
| 9 | P10/2 | sensitive to the [inflammable](http://dict.cnki.net/dict_result.aspx?searchword=易燃&tjType=sentence&style=&t=inflammable) gas |
| 10 | P40/1 | sensitive to the strong oxidizing gas |
| 11 | T70/2 | sensitive to the [aromatic compounds](http://dict.cnki.net/dict_result.aspx?searchword=芳香族化合物&tjType=sentence&style=&t=aromatic+compounds) |
| 12 | PA/2 | sensitive to the [organic compounds](http://dict.cnki.net/dict_result.aspx?searchword=有机化合物&tjType=sentence&style=&t=organic+compounds) and toxic gas |
| 13 | P30/1 | sensitive to the [combustible](http://dict.cnki.net/dict_result.aspx?searchword=可燃&tjType=sentence&style=&t=combustible) gas and [organic compounds](http://dict.cnki.net/dict_result.aspx?searchword=有机化合物&tjType=sentence&style=&t=organic+compounds) |
| 14 | P40/2 | sensitive to the strong oxidizing gas |
| 15 | P30/2 | sensitive to the [organic compounds](http://dict.cnki.net/dict_result.aspx?searchword=有机化合物&tjType=sentence&style=&t=organic+compounds) |
| 16 | T40/2 | sensitive to the strong oxidizing gas |
| 17 | T40/1 | sensitive to the strong oxidizing gas |
| 18 | TA/2 | sensitive to the [organic compounds](http://dict.cnki.net/dict_result.aspx?searchword=有机化合物&tjType=sentence&style=&t=organic+compounds) |

**2.2** **The** **results of** **electronic nose test**

The statistical analysis of the data collected by electronic nose test was handled according to the maximum response intensity of the sensors.

**Table 3 The results of electronic nose test**

|  | *Jinzhen* | Placebo |
| --- | --- | --- |
| LY2/LG | 0.143221 | 0.083245 |
| LY2/G | -0.601855 | -0.397203 |
| LY2/AA | -0.686195 | -0.483426 |
| LY2/GH | -0.491672 | -0.320878 |
| LY2/gCTl | -0.461320 | -0.277183 |
| LY2/gCT | -0.084140 | -0.049504 |
| T30/1 | 0.553840 | 0.433720 |
| P10/1 | 0.466067 | 0.359101 |
| P10/2 | 0.362380 | 0.284171 |
| P40/1 | 0.364279 | 0.279197 |
| T70/2 | 0.462669 | 0.318280 |
| PA/2 | 0.635752 | 0.484630 |
| P30/1 | 0.792981 | 0.659887 |
| P40/2 | 0.567629 | 0.462334 |
| P30/2 | 0.552436 | 0.418360 |
| T40/2 | 0.289547 | 0.217373 |
| T40/1 | 0.191286 | 0.137705 |
| TA/2 | 0.188432 | 0.151135 |
| Similarity | 91.60% | |

The results showed that the similarity of the two testing samples in smell is 91.60%.

**3 Electronic tongue test**

**3.1 Performance of the sensors**

There are 7 sensors in electronic tongue. These sensors are all made from metal oxide. The names and performances of these sensors are shown as Table 4.

Table 4 Performance of the sensors in electronic tongue

|  |  | ZZ | BA | BB | CA | GA | HA | JB | Sensor Set#1threshold | Human threshold |
| --- | --- | --- | --- | --- | --- | --- | --- | --- | --- | --- |
| Sour | Acetic acid | 10^-7^ | 10^-6^ | 10^-7^ | 10^-7^ | 10^-7^ | 10^-7^ | 10^-7^ | 10^-7^ | 1.1*10^-4^ |
|  | Citric acid | 10^-7^ | 10^-6^ | 10^-7^ | 10^-7^ | 10^-7^ | 10^-6^ | 10^-6^ | 10^-7^ | 7*10^-5^ |
|  | HCl | 10^-7^ | 10^-7^ | 10^-7^ | 10^-7^ | 10^-7^ | 10^-7^ | 10^-7^ | 10^-7^ | 1.6*10^-4^ |
|  | Malic acid | 10^-6^ | 10^-4^ | 10^-6^ | 10^-6^ | 10^-5^ | 10^-5^ | 10^-5^ | 10^-6^ | 7.3*10^-5^ |
|  | Tartari acid | 10^-6^ | 10^-5^ | 10^-5^ | 10^-5^ | 10^-5^ | 10^-5^ | 10^-5^ | 10^-5^ | 4.78*10^-5^ |
| Salt | NH_4_Cl | 10^-6^ | 10^-4^ | 10^-6^ | 10^-6^ | 10^-4^ | 10^-4^ | 10^-4^ | 10^-6^ | 8.4*10^-4^ |
|  | CaCl_2_ | 10^-7^ | 10^-7^ | 10^-7^ | 10^-5^ | 10^-7^ | 10^-4^ | 10^-5^ | 10^-7^ | 8*10^-6^ |
|  | LiCl | 10^-7^ | 10^-7^ | 10^-7^ | 5*10^-5^ | 10^-7^ | 10^-5^ | 10^-5^ | 10^-7^ | 2*10^-2^ |
|  | NaCl | 10^-6^ | 10^-5^ | 10^-6^ | 10^-6^ | 10^-4^ | 10^-4^ | 10^-5^ | 10^-6^ | 10^-3^ |
|  | KCl | 10^-7^ | 10^-4^ | 10^-4^ | 10^-5^ | 10^-4^ | 10^-4^ | 10^-4^ | 10^-4^ | 6.4*10^-3^ |
| Sweet | Aspartame | 10^-7^ | 10^-7^ | 10^-7^ | 10^-5^ | 10^-7^ | 10^-5^ | 10^-5^ | 10^-7^ | 1.9*10^-5^ |
|  | Fructose | 10^-5^ | 10^-4^ | 10^-5^ | 10^-5^ | 10^-5^ | 10^-4^ | 10^-5^ | 10^-5^ | 8.9*10^-4^ |
|  | Glucose | 10^-7^ | 10^-4^ | 10^-7^ | 10^-7^ | 10^-4^ | 10^-4^ | 10^-4^ | 10^-7^ | 7.3*10^-3^ |
|  | Glycine | 10^-7^ | 10^-7^ | 10^-7^ | 5*10^-5^ | 10^-7^ | 10^-3^ | 10^-3^ | 10^-7^ | 3.1*10^-2^ |
|  | Saccharin.Na | 10^-6^ | 10^-4^ | 10^-6^ | 10^-6^ | 10^-4^ | 10^-4^ | 10^-4^ | 10^-6^ | 9.2*10^-6^ |
|  | Sucrose | 10^-5^ | 10^-4^ | 10^-5^ | 10^-5^ | 10^-4^ | 10^-4^ | 10^-4^ | 10^-5^ | 6.5*10^-4^ |
| Bitter | Caffeine | 10^-5^ | 10^-4^ | 10^-4^ | 10^-5^ | 10^-4^ | 10^-4^ | 10^-4^ | 10^-4^ | 5*10^-4^ |
|  | MgSO_4_ | 10^-6^ | 10^-4^ | 10^-5^ | 10^-6^ | 10^-5^ | 10^-4^ | 10^-4^ | 10^-5^ | 3.8*10^-4^ |
|  | Quinine.HCl | 10^-4^ | 10^-4^ | 10^-4^ | 10^-4^ | 10^-4^ | 10^-4^ | 10^-4^ | 10^-4^ | 1.4*10^-6^ |
|  | Urea | 10^-7^ | 10^-7^ | 10^-7^ | 10^-7^ | 10^-6^ | 10^-6^ | 10^-6^ | 10^-6^ | 1.5*10^-4^ |
| Savoury | MSG | 10^-5^ |  | 10^-4^ | 10^-5^ | 10^-4^ | 10^-4^ | 10^-4^ | 10^-4^ | 5*10^-4^ |
|  | L-arginine | 10^-6^ | 10^-4^ | 10^-6^ | 10^-5^ | 10^-5^ | 10^-4^ | 10^-5^ | 10^-5^ | 1.2*10^-3^ |
|  | L-glutamine | 5*10^-4^ | 5*10^-4^ | 5*10^-3^ | 5*10^-3^ | 5*10^-3^ | 5*10^-3^ | 5*10^-3^ | 5*10^-3^ | 9.8*10^-3^ |

**3.2** **The results of electronic tongue test**

The statistical analysis of the data collected by electronic nose test was handled according to the maximum response intensity of the sensors.

**Table 5 The results of electronic tongue test**

|  | *Jinzhen* | Placebo |
| --- | --- | --- |
| ZZ | 1482.481 | 1794.903 |
| BA | -3765.39 | -3496.52 |
| BB | -3895.36 | -3510.02 |
| CA | -2768.32 | -2708.22 |
| GA | -2794.26 | -2720.58 |
| HA | -4882.21 | -4766.65 |
| JB | -3786.44 | -3694.3 |
| Similarity | 90.86% | |

The results showed that the similarity of the two testing samples in taste is 90.86%.

**4** **The comprehensive similarity evaluation of the comparison between *Jinzhen* oral liquid and the placebo**

The comprehensive similarity evaluation of the comparison between *Jinzhen* oral liquid and the placebo was performed with the weight coefficients of 50% in visual appearance, 20% in smell and 30% in taste. The results were shown in Table 6.

**Table 6 The result of the** **comprehensive similarity evaluation**

|  | **machine vision**（50%） | **electronic nose**（20%） | **electronic tongue**（30%） | **comprehensive similarity** |
| --- | --- | --- | --- | --- |
| **Similarity** | 95.27% | 91.60% | 90.86% | 93.225% |

The results showed that the comprehensive similarity of the two testing samples was 93.225%.

**5 Conclusions**

According to the results from the comparative evaluation of Intelligent sensory technology including [machine vision](dict://key.0895DFE8DB67F9409DB285590D870EDD/machine vision), electronic nose and electronic tongue technique, the comprehensive similarity of *Jinzhen* oral liquid and the placebo is 93.225%. The placebo is the matched one of *Jinzhen* oral liquid in visual appearance, smell and taste.
