# Supplementary material for: Therapeutic Effect of Jinzhen Oral Liquid for Hand Foot and Mouth Disease: A Randomized, Multi-Center, Double-Blind, Placebo-Controlled Trial
Source: PLoS One. 2014 Apr 10;9(4):e94466. doi: 10.1371/journal.pone.0094466 (PMC3983189; doi:10.1371/journal.pone.0094466)
Supplement: Protocol S1 — The protocol of “ Jinzhen oral liquid for Hand Foot and Mouth Disease in Children: a Randomized, Multi-center, Double-blind, Placebo-controlled Trial”. (DOC) [file pone.0094466.s004.doc]

## Protocol S1

## Protocol

***Jinzhen* oral liquid for Hand Foot and Mouth Disease in Children: a Randomized, Multi-center, Double-blind, Placebo-controlled Trial**

**First Draft: May 25th 2010**

**Updated: June 24^th^ 2010**

**Sponsored by:**

Traditional Chinese Medicine (TCM) Research Project of TCM Preventing and Curing Infectious Diseases of State Administration of TCM of People’s Republic of China (200907001-5)

**Complications Scientific Committee Chair:** Prof. Yongyan Wang

**Protocol Co-Chairs:** Prof. Yong-Yan Wang; Prof. Zhong Wang, Ph.D.; Prof. Guo-Liang Zhang; Wei Xiao, Ph.D

**Protocol Vice Chairs:** Prof. Gui-Qin Huang; Li Li; Chun-Ping Li; Prof. Hao Yu, Ph.D

**Clinical Trial** **Specialists:** Jun Liu, Ph.D; Ya-Nan Yu; Prof. Li-Yun He, M.D.

Shi-Yan Yan, Ph.D

**PARTICIPATING SITES**

**Infectious Disease Hospital of Cangzhou City,** No.68 Guangrong Road, Cangzhou, 061001, China;

**The Fifth People’s Hospital of Guiyang City,** No.2 Nanduan.Xintian Road, Guiyang, 550004, China

**Infectious Disease Hospital of Tangshan City,** No.11 Changning Road, Tangshan, 063020, China

**PROTOCOL TEAM ROSTER**

**Protocol Co-Chairs**

**Prof. Yong-Yan Wang**

**Co-Director,** **Institute of Basic Research in Clinical Medicine**, China Academy of Chinese Medical Sciences, No. 16 Nanxiaojie, Dongzhimen nei, Beijing, 100700, China

Tel: **86-10-84046033,** E-mail: wangyongyan2010@sina.cn

**Prof.** **Zhong Wang, Ph. D**

**Institute of Basic Research in Clinical Medicine**, China Academy of Chinese Medical Sciences, No.16 Nanxiaojie, Dongzhimen nei, Beijing, 100700, China

Tel: 86-10-64014411-2805, E-mail: zhonw@vip.sina.com

**Prof.** **Guo-Liang Zhang**

**Infective Disease Department,** First Affiliated Hospital of Anhui University of Traditional Chinese Medicine, No.117 Meishan Road, Hefei, 230031, China

Tel: 86-0-13905607286, E-mail: glzhang_anhui@sina.com

**Wei Xiao, Ph.D.**

**Kanion Pharmaceutical Company**, No.58 Haichang South Road, Xinpu District, Lianyungang, 222001, China.

E-mail: xw@kanion.com

**Protocol Vice Chairs**

**Prof.** **Gui-Qin Huang**

**Infectious Disease Hospital of Cangzhou City**, No.68 Guangrong Road, Cangzhou, 061001, China

E-mail: hgq_cangzhou@sina.com

**Li Li**

**The Fifth People’s Hospital of Guiyang City,** No.2 Nanduan.Xintian Road,Guiyang, 550004, China

E-mail: liligy67@163.com

**Chun-Ping Li**

**Infectious Disease Hospital of Xian’an City,** No.11 Changning Road, Xian’an, Tangshan, 063020, China

E-mail: lichunping-ts@sohu.com

**Prof. Hao Yu, Ph.D.**

**Department of Epidemiology and Biostatistics**, Nanjing Medical University, No.140 Hanzhong Road, Nanjing, 210029,China

Email: njyuhao@vip.sina.com

**Prof. Li-Yun He, M.D., Ph.D.**

**Institute of Basic Research in Clinical Medicine**, China Academy of Chinese Medical Sciences, No. 16 Nanxiaojie, Dongzhimen nei, Beijing, 100700, China

E-mail: hely3699@163.com

**Clinical Trial Specialists**

**Jun Liu, Ph. D**

**Institute of Basic Research in Clinical Medicine**, China Academy of Chinese Medical Sciences, No. 16 Nanxiaojie, Dongzhimen nei, Beijing, 100700, China

E-mail: franlj1104@aliyun.com

**Ya-Nan Yu**

**Institute of Basic Research in Clinical Medicine**, China Academy of Chinese Medical Sciences, No. 16 Nanxiaojie, Dongzhimen nei, Beijing, 100700, China

E-mail: pumpkinnaicha@163.com

**Cangzhou Investigators**

**Xiu-Rong Sun**

**Infectious Disease Hospital of Cangzhou City**, No.68 Guangrong Road, Cangzhou, 061001, China

Email: sunxiurong1981@sohu.com

**Hong-Sen Zhang**

**Infectious Disease Hospital of Cangzhou City,** No.68 Guangrong Road, Cangzhou, 061001, China

Email: zhanghs7677@126.com

**Guiyang Investigators**

**Mei Wang**

**The Fifth People’s Hospital of Guiyang City,** No.2 Nanduan.Xintian Road, Guiyang, 550004, China

Email: wangmeiwm57@163.com

**Xiao-Yan Liang**

**The Fifth People’s Hospital of Guiyang City,** No.2 Nanduan.Xintian Road, Guiyang, 550004, China

Email: lxygy1963@163.com

**Di Xie**

**The Fifth People’s Hospital of Guiyang City,** No.2 Nanduan.Xintian Road, Guiyang, 550004, China

Email: xiedi1979@163.com

**Chang-Ming Yang**

**The Fifth People’s Hospital of Guiyang City**, No.2 Nanduan.Xintian Road, Guiyang, 550004, China

Email: ycmgy1956@163.com

**Yan Li**

**The Fifth People’s Hospital of Guiyang City,** No.2 Nanduan.Xintian Road, Guiyang, 550004, China

Email: liyan1975@163.com

**Tangshan Investigators**

**Bai-Song Wan**

**Infectious Disease Hospital of Xian’an City,** No.11 Changning Road, Xian’an, Tangshan, 063020, China

Email: baisongwants@sohu.com

**Wei-Hua Zhang**

**Infectious Disease Hospital of Xian’an City,** No.11 Changning Road, Xian’an, Tangshan, 063020, China

Email: lantianweihua521@sohu.com

**Protocol Statisticians**

**Prof.** **Hao Yu,** **Ph.D.**

**Department of Epidemiology and Biostatistics**, Nanjing Medical University, No.140 Hanzhong Road, Nanjing, 210029, China

Email: njyuhao@vip.sina.com

**Ru-Yang Zhang, Ph.D.**

**Department of Epidemiology and Biostatistics,** Nanjing Medical University, No.140 Hanzhong Road, Nanjing, 210029, China

Email: zhangruyang2008@126.com

**Protocol Data Managers**

**Prof. Li-Yun He, M.D., Ph.D.**

**Institute of Basic Research in Clinical Medicine**, China Academy of Chinese Medical Sciences, No. 16 Nanxiaojie, Dongzhimen nei, Beijing, 100700, China

E-mail: hely3699@163.com

**Shi-Yan Yan, Ph. D**

**Institute of Basic Research in Clinical Medicine**, China Academy of Chinese Medical Sciences, No. 16 Nanxiaojie, Dongzhimen nei, Beijing, 100700, China

E-mail: yanshiyan0927@sina.com

**Protocol Pharmacists**

**Wei Xiao****, Ph.D.**

**Kanion Pharmaceutical Company**, No.58 Haichang South Road, Xinpu District, Lianyungang, 222001, China.

E-mail: xw@kanion.com

**Zhen-Zhong Wang, Ph.D.**

**Kanion Pharmaceutical Company**, No.58 Haichang South Road, Xinpu District, Lianyungang, 222001, China.

E-mail: wzhzh-nj@tom.com

**Site Study Coordinators**

**Suliya**

**Institute of Basic Research in Clinical Medicine**, China Academy of Chinese Medical Sciences, No. 16 Nanxiaojie, Dongzhimen nei, Beijing, 100700, China

Tel: 86-0-15101016441

**Hong-Li Wu, Ph. D**

**Institute of Basic Research in Clinical Medicine**, China Academy of Chinese Medical Sciences, No. 16 Nanxiaojie, Dongzhimen nei, Beijing, 100700, China

Tel: 86-0-13716165325; E-mail: thisyear2006@126.com

CONTENTS

[**TITLE PAGE 1**](#__RefHeading___Toc303262667)

[**PARTICIPATING SITES 2**](#__RefHeading___Toc303262668)

[**PROTOCOL TEAM ROSTER 2**](#__RefHeading___Toc303262669)

[**GLOSSARY 10**](#__RefHeading___Toc303262670)

[**SUMMARY OF CHANGES 12**](#__RefHeading___Toc303262671)

[**SCHEMA 13**](#__RefHeading___Toc303262672)

[**1. BACKGROUND INFORMATION 15**](#__RefHeading___Toc303262673)

[**2. STUDY RATIONALE 18**](#__RefHeading___Toc303262674)

[**3. OBJECTIVES 19**](#__RefHeading___Toc303262675)

[**3.1 Primary objective 19**](#__RefHeading___Toc303262676)

[**3.2 Second objectives 19**](#__RefHeading___Toc303262677)

[**4. SELECTION AND ENROLLMENT OF STUDY PARTICIPANTS 19**](#__RefHeading___Toc303262678)

[**4.1 Diagnostic Criteria 19**](#__RefHeading___Toc303262679)

[**4.2 Inclusion Criteria 20**](#__RefHeading___Toc303262680)

[**4.3 Exclusion Criteria 20**](#__RefHeading___Toc303262681)

[**4.4 Criteria for Treatment Discontinuation 20**](#__RefHeading___Toc303262682)

[**4.5 Criteria for Treatment and Study Discontinuation 21**](#__RefHeading___Toc303262683)

[**5. STUDY DESIGN 21**](#__RefHeading___Toc303262684)

[**5.1 Summary of Study Design 21**](#__RefHeading___Toc303262685)

[**5.2 Randomization and Allocation Concealment 21**](#__RefHeading___Toc303262686)

[**5.3 Cases Assignment 22**](#__RefHeading___Toc303262687)

[**5.4 Blinding 22**](#__RefHeading___Toc303262688)

[**6. STUDY TREATMENT AND FOLLOW-UP 23**](#__RefHeading___Toc303262689)

[**6.1 Preparation Quality Control 23**](#__RefHeading___Toc303262690)

[**6.2 Treatment Regimens 23**](#__RefHeading___Toc303262691)

[**6.3 Duration and Follow-up 25**](#__RefHeading___Toc303262692)

[**7. OBSERVATION INFORMATION 26**](#__RefHeading___Toc303262693)

[**7.1 Baseline 26**](#__RefHeading___Toc303262694)

[**7.2 Observation Parameters 27**](#__RefHeading___Toc303262695)

[**8. OUTCOME MEASURES 28**](#__RefHeading___Toc303262696)

[**8.1 Primary outcome measures 28**](#__RefHeading___Toc303262697)

[**8.2 Secondary outcome measures 28**](#__RefHeading___Toc303262698)

[**9 SAFETY INSTRUCTIONS AND GUIDANCE 29**](#__RefHeading___Toc303262699)

[**9.1 Previous safety information 29**](#__RefHeading___Toc303262700)

[**9.2 Adverse Events (AEs) and Laboratory Abnormalities 30**](#__RefHeading___Toc303262701)

[**9.3 Handling of Safety Parameters 32**](#__RefHeading___Toc303262702)

[**10. DATA COLLECTION, MANAGEMENT AND QUALITY ASSURANCE 34**](#__RefHeading___Toc303262703)

[**10.1 Collection and Fulfillment of CRF 34**](#__RefHeading___Toc303262704)

[**10.2 Data Collection and management 34**](#__RefHeading___Toc303262705)

[**10.3 Data Lock 34**](#__RefHeading___Toc303262706)

[**11. STASTISTICAL CONSIDERATIONS AND ANALYTICAL PLAN 35**](#__RefHeading___Toc303262707)

[**11.1 Sample Size Calculation 35**](#__RefHeading___Toc303262708)

[**11.2 Analysis Sets 35**](#__RefHeading___Toc303262710)

[**11.3 Statistical Methods 36**](#__RefHeading___Toc303262711)

[**11.4 Other Statistical Considerations 38**](#__RefHeading___Toc303262712)

[**12. ETHICAL ASPECTS 38**](#__RefHeading___Toc303262713)

[**12.1 Local Regulations/Declaration of Helsinki 38**](#__RefHeading___Toc303262714)

[**12.2 Informed Consent 38**](#__RefHeading___Toc303262715)

[**12.3 Institutional Review Board (IRB) 39**](#__RefHeading___Toc303262716)

[**13. CONDITIONS FOR MODIFYING THE PROTOCOL 40**](#__RefHeading___Toc303262717)

[**14. CONDITIONS FOR TERMINATING THE STUDY 40**](#__RefHeading___Toc303262718)

[**15. STUDY DOCUMENTATION, CRFS AND RECORD KEEPING 40**](#__RefHeading___Toc303262719)

[**15.1 Investigator's Files / Retention of Documents 40**](#__RefHeading___Toc303262720)

[**15.2 Source Documents and Background Data 42**](#__RefHeading___Toc303262721)

[**15.3 Audits and Inspections 43**](#__RefHeading___Toc303262722)

[**15.4 Case Report Forms 43**](#__RefHeading___Toc303262723)

[**16. MONITORING THE STUDY 43**](#__RefHeading___Toc303262724)

[**17. CONFIDENTIALITY OF TRIAL DOCUMENTS AND SUBJECT RECORDS 44**](#__RefHeading___Toc303262725)

[**18. PUBLICATION OF DATA AND PROTECTION OF TRADE SECRETS 44**](#__RefHeading___Toc303262726)

[**19. REFERENCES 45**](#__RefHeading___Toc303262727)

[**APPENDICES 49**](#__RefHeading___Toc303262728)

[**Appendix 1 Fingerprint electropherogram and chemical structures of the *Jinzhen* oral liquid 49**](#__RefHeading___Toc303262729)

[**Appendix 2. Adverse Events Categories for Determining Relationship to Test Drug** **51**](#__RefHeading___Toc303262731)

[**Appendix 3. ICH Guidelines for Clinical Safety Data Management, Definitions and Standards for Expedited Reporting, Topic E2** **53**](#__RefHeading___Toc303262732)

**GLOSSARY**

AE adverse event

CoxA16 Coxsackie group A 16

CRF case report form

CRS central randomization system

DQF data queries form

ECG electrocardiogram

EV71 enterovirus 71

FAS full analysis set

GCP Good Clinical Practice

HFMD hand foot and mouth disease

HPLC high-performance liquid chromatography

IC 50 Half-inhibitory Concentration

ICH International Conference of Harmonisation

ICF informed consent form

IRB Institutional Review Board

ITT intention-to-treat

IVR interactive voice response

IWR interactive web response

NIH National Institutes of Health

PPS per protocol set

RT-PCR real time polymerase chain-reaction

SAE serious adverse event

SDV source data verification

SFDA State Food and Drug Administration

SS safety analysis set

TCM traditional Chinese medicine

US United States

US CDC Center for Disease Control of the United States

**SUMMARY OF CHANGES**

**Summary of Changes for ChiCTR-TRC-10000937, *Jinzhen* oral liquid for Hand Foot and Mouth Disease in Children: A Pragmatic, Double-blind Randomized Controlled Trial**

**All changes in this version appear in boldface type. Major changes include the following:**

**1) In Section 1 “BACKGROUND INFORMATION”, we updated the information about the epidemic of HFMD according to the news from Ministry of Health of the People’s Republic of China. (Updated on June 24^th^ 2010)**

**2)** **In Section 8.2.2, we changed the secondary outcome measure “the incidence of severe case” into “treatment failure rate”, since there is not an agreement to the definition of “severe case” nowadays. (Updated on June 9^th^ 2010)**

**3) In Section 8.2.5, according to the** **Institutional Review Board (IRB), it’s very difficult to calculate the direct economic cost of the treatment in China, so in the final edition of the protocol, we removed this measure. (Updated on June 5^th^ 2010)**

**SCHEMA**

***Jinzhen* oral liquid for Hand Foot and Mouth Disease in Children:** **A Pragmatic, Double-blind Randomized Controlled T**rial

| DESIGN:  pragmatic, multicenter, randomized, placebo-controlled, double-blind |
| --- |
| SAMPLE SIZE:  Total: 460 study participants with HFMD |
| POPULATION:  Children aged 1-7 years who fulfill the diagnostic criteria for HFDM will be enrolled with a history of fever or vesicles with a maximum duration of 48 hrs and a temperature no more than 39℃ |
| RANDOMIZATION：  Randomization to *Jinzhen* or its placebo will be in a 1:1 ratio |
| REGIMEN:  *Jinzhen* or placebo (1ml/kg/ dose orally, three times a day); when the child’s temperature exceeds 38.5℃, ibuprofen suspension (Motrin, Johnson & Johnson) will also be administered according to the label dosage. |
| TREATMENT DURATION:  Study participants will receive *Jinzhen* or placebo for 7 days and will be followed on study for an additional 3 days (a total of 10 days since the time of study enrollment) |
| OUTCOME MEASURES:  Primary:  1. Time to the first disappearance of oral ulcers and vesicles on hand or foot within 10 days (including 3-day follow up);  2. Time to the first normalization of temperature (fever clearance) including time to the first drop of temperature greater than 0.5℃ at 3, 6, 12 and 24 hrs after treatment.  Secondary:  1. The proportion of children whose symptoms disappear within 10 days (assessed daily);  2. The frequency and dose of ibuprofen, if used;  3. **Treatment failure rate;**  4. Safety profiles: including a description of all unanticipated events and undesirable experiences, particularly exacerbations of HFMD symptoms. |

**1. BACKGROUND INFORMATION**

HFMD is a highly contagious common childhood viral disease, which is usually caused by Coxsackie group A (CoxA16) enteroviruses or enterovirus 71 (EV71). The disease causes fever, oral ulcers, vesicles on hands, feet and buttocks, and may further develop into myocarditis, pulmonary edema, aseptic meningoencephalitis, and other complications [1](http://www.ncbi.nlm.nih.gov/pmc/articles/PMC3079592/?tool=pubmed#B2),[2](http://www.ncbi.nlm.nih.gov/pmc/articles/PMC3079592/?tool=pubmed#B3). In the past three decades, several severe outbreaks of HFMD took place throughout the world, especially in the Asia-Pacific region^3^. The epidemic of HFMD that was associated with EV71 infection occurred in Taiwan in 1998, leading to 129,106 cases of HFMD and 405 severe cases with complications^4^. This epidemic resulted in 78 deaths, most of which were caused by cardiopulmonary failure^5^, and was followed by outbreaks in other parts of the Asia-Pacific region, including Singapore, Australia and Japan^6.7^. The recent major epidemic in the mainland of China involved 488,955 reported cases during the year 2008, with a morbidity of 37/100,000, a mortality of 0.0095/100,000 and ill-death rate 0.26/1000, as well as 1,155,525 cases during 2009, and **by the end of June 24, 2010, 987,779 cases of HFMD, resulting in15,501 cases of severe complication and 537 deaths**^8^.

HFMD usually resolves spontaneously, although it can be life threatening if the viruses cause inflammation of the brain stem, which can progress to heart failure and pulmonary edema. However, there is as yet no effective vaccine to prevent its infection or no specific antiviral agent against this disease. Supportive care or symptomatic therapy is all that available at present. Moreover, according to the PubMed, only two trials of supportive treatment have been reported for HFMD: one was a randomized, one-center, double-blind, placebo-controlled trial of patients with HFMD-induced painful stomatitis treated with low-lever laser^9^; and the other was a historically controlled trial of 24 children with severe EV71-induced pulmonary edema treated with milrinone^10^.

A therapeutic approach that targets several sites of pathobiology and pathway pathology has been considered promising and already proven to be successful in the treatment of a variety of infective diseases, including SARS^11^, epidemic influenza H1N1^12^, and lethal sepsis^13^. HFMD is caused by numerous members of the Enterovirus genus e.g. Cox A and EV71, which may induce multiple system organ damage.

As we know, the millennia-old traditional Chinese medicine (TCM) can treat certain infective diseases with many combination regimens involving some ingredients commonly used in clinical practice, which have been proved to have antipyretic^14^, anti-inflammatory^15^, and antiviral activities^16, 17^, especially the antiviral activity against EV71 and coxsackievirus^18^. According to its clinical and epidemiological characteristics, HFMD belongs to the epidemic febrile disease of traditional Chinese medicine. Ministry of Health of the People's Republic of China issued "Diagnosis and treatment guidelines of HFMD (2010 edition)"(available at <http://www.moh.gov.cn/publiles/business/htmlfiles/mohyzs/s3586/201004/46884.htm>, Accessed on April 22^th^ 2010), which recommended several Chinese patent medicines for HFMD treatment, such as *Lanqin* oral liquid, *Xiaoerchiqiao qingre* granules, *kangbingdu* oral liquid, etc. However, there was insufficient evidence to support their widespread clinical use in the treatment of HFMD.

*Jinzhen* oral liquid, approved as a Chinese patent medicine by State Food and Drug Administration (SFDA) of China, has been recorded into Pharmacopoeia of the People's Republic of China in 2010 ^18^ (its fingerprint electropherogram and the chemical structure of the major bioactive compounds in *Jinzhen* shown in **Appendix 1**). *Jinzhen* oral liquid roots in a traditional popular prescription "Lingyang Qingfei San", a remedy for cooling blood to remove apthogenic heat which has been used as a basic formula in traditional Chinese medicine in treating children's illnesses for many years. The formula is composed of eight traditional Chinese medicinal materials native to Mainland China: ussuri fritillary bulb (*Pingbeimu*), rhubarb (*Dahuang*), baikal skullcap root (*Huangqin*), biotite schist (*Qingmengshi*), gypsum (*Shigao*), artificial bezoar (*Rengong Niuhuang*), and Liquorice Root (*Gancao*). The major bioactive ingredients in this oral liquid have been demonstrated to have some active pharmacological functions, e.g. baicaline have antipyretic activities^20^; Rheum tanguticum polysaccharide extracted from rhubarb may provide immunoregulatory and anti-inflammatory effects for inflammatory diseases^21^; Baicaline inhibits IL-1β- and TNF-α-induced inflammatory cytokine^22^ and inhibits inflammatory cyclooxygenase-2 gene expression^23^, which possesses antiviral and anti-inflammatory properties^24^; Roasted licorice extracts may exert anti-inflammatory effect on lipopolysaccharide-induced inflammatory reactions in murine macrophages^25^; and Glycyrrhizin, an active component of licorice roots, is effective in reducing the morbidity and mortality of mice infected with lethal doses of influenza virus^26^. Moreover, although the results haven’t been published, before starting the trial, we conducted two antiviral experiments of *Jinzhen* oral liquid in vitro which indicated that *Jinzhen* oral liquid could inhibit replication of EV71 and Cox A16 with the estimated half-inhibitory concentration (IC 50) value of 4.1mg/mL and 2.3mg/mL, respectively. A pilot clinical study conducted in 42 children with HFMD, which has not been published, showed that compared with using ribavirin alone, *Jinzhen* oral liquid combined with ribavirin could both reduce the duration of fever and the time to the first disappearance of oral ulcers and vesicles on hand or foot.

We plan to conduct a double-blind, randomized, placebo-controlled trial to assess the antipyretic and anti-inflammatory benefits of *Jinzhen* compared with placebo. We hypothesize that at the end of the 7-day intervention period and 3-day follow-up, patients in the *Jinzhen* group would have greater reductions in time to the first normalization of temperature (fever clearance), time to the first disappearance of oral ulcers and vesicles on hand or foot, and treatment failure rate, as well as greater improvements in clinical symptoms as compared with the placebo group.

In our study, the quality control of ingredients in *Jinzhen* oral liquid will be determined by high-performance liquid chromatography (HPLC) fingerprinting, while the placebo and treatment medication will be all manufactured in a same batch. Therefore, the pharmacological effects of *Jinzhen* oral liquid and placebo will be relatively stable in the trial.

**2. STUDY RATIONALE**

2.1 The bioactive function of the compounds in *Jinzhen* oral liquid

2.2 The pharmacological effect and the previous clinical use of *Jinzhen* oral liquid for children with fever according to its label

2.3 Severe outbreaks of HFMD in China in 2009-2010

2.4 Use of *Jinzhen* placebo

This trial will administer study treatment in a double-blind, placebo-controlled manner (1:1). Neither the family nor the medical care team will be able to identify who is receiving *Jinzhen*. All children who develop HFMD will be treated as per the “Diagnosis and treatment guidelines of HFMD (2010 edition)” issued by Ministry of Health of China(available at http://www.moh.gov.cn/publicfiles/business/htmlfiles/mohyzs/s3586/201004/46884.htm). *Jinzhen* oral liquid and its placebo will be controlled by HPLC in order to ensure the consistency of study drugs, and placebo will not continue to be given to the children who develop to severe cases such as encephalitis in the trial.

**3. OBJECTIVES**

3.1 Primary objective

To determine the efficacy of *Jinzhen* oral liquid versus placebo in reducing the time to the first disappearance of oral ulcers and vesicles on hand or foot and the time to the first normalization of temperature (fever clearance).

3.2 Second objectives

3.2.1 To assess the efficacy of *Jinzhen* oral liquid in reducing signs and symptoms of HFMD;

3.2.2 To investigate whether *Jinzhen* oral liquid can reduce the frequency and dose of ibuprofen in those children with a temperature≥38.5 ℃;

**3.2.3 To assess the effect of *Jinzhen* oral liquid in decreasing the treatment failure rate;**

3.2.4 To assess the safety of *Jinzhen* oral liquid during the treatment for HFMD.

**4. SELECTION AND ENROLLMENT OF STUDY PARTICIPANTS**

4.1 Diagnostic Criteria

According to the “Diagnosis and treatment guidelines of HFMD (2010 edition)” issued by Ministry of Health of China (available at http://www.moh.gov.cn/publicfiles/business/htmlfiles/mohyzs/s3586/201004/46884.htm, accessed on April 22^th^ 2010), HFMD is defined as blister-like oral ulcers and vesicles on hand or foot with or without buttock involvement, accompanied or not accompanied by fever.

A HFMD case will be considered confirmed with a positive laboratory results. The samples from the throat or stool will be sent to the laboratory of Chinese Centre for Disease Control and Prevention to test for virus and to find out which enterovirus caused the illness. The non-polio enteroviruses-infection status of children will be determined by means of non-polio enteroviruses (mainly as EV71 and Cox A16) real time polymerase chain-reaction (RT-PCR) testing.

4.2 Inclusion Criteria

4.2.1 Children who fulfill the diagnostic criteria of HFDM;

4.2.2 Aged 1 to 7 years;

4.2.3 Those with a history of fever or vesicles with a maximum duration of 48 hrs;

4.2.4 Those with a temperature ≤39℃;

4.2.5 Parent or legal guardian able and willing to provide signed informed consent and anticipate residence in hospital for at least 10 days.

4.3 Exclusion Criteria

4.3.1 Those children who are complicated with other serious diseases, such as congenital heart disease, chronic hepatitis, nephritis, hematological diseases, etc;

4.3.2 Those who are prone to allergies or with known allergy to the study drugs;

4.3.3 Those or their guardians with unstable psychiatric disorders;

4.3.4 Those with chronic diarrhea;

4.3.5 Those who are participating in other clinical trials.

4.4 Criteria for Treatment Discontinuation

Study participants will be discontinued from study treatment but remain on study for any of the following reasons. The study participant will be treated per country specific standard of care.

Study participants should complete study visits and laboratory evaluations scheduled.

4.4.1 The investigator determines that further participation would be detrimental to the study participant's health or well being.

4.4.2 The study participant requires treatment with medications that are disallowed while on study,

4.4.3 The study participant experiences drug toxicity necessitating study drug discontinuation as defined in Section 9.2

4.4.4 The study participant develops severe cases, such as myocarditis, encephalitis, aseptic meningitis, pulmonary edema, and poliomyelitis-like paralysis.

4.5 Criteria for Treatment and Study Discontinuation

Study participants will be discontinued from study treatment and go off study for any of the following reasons.

Study participants should complete an off study form and all study laboratory evaluations as per day 7.

4.5.1 The parent/legal guardian refuses further treatment and/or follow-up evaluations;

4.5.2 The HFMD study participant takes medication less than three times.

**5. STUDY DESIGN**

5.1 Summary of Study Design

This is a multicenter, randomized, placebo-controlled, double blind trial to evaluate the efficacy and safety of *Jinzhen* oral liquid for HFMD.

5.2 Randomization and Allocation Concealment

Stratified blocked randomization will be conducted in our trial based on the site. The random numbers are generated via PROC PLAN process of SAS 9.1.3. We will assign participants to the *Jinzhen* or placebo intervention with 1:1 ratio in two randomization cycles, using computer-generated numbers. The randomized treatment assignments are managed by central randomization system (CRS) via interactive web response (IWR) and a randomization number is assigned individually for each patient whose parent or guardian agrees to participate in the study. Parents, investigators, and statisticians are all blinded to treatment allocation by using identically matched placebos. While the IWR cannot work, the interactive voice response (IVR) will be conducted instead.

5.3 Cases Assignment

This trial is undertaken in 3 centers across China: The Fifth People’s Hospital of Guiyang City, Infectious Disease Hospital of Cangzhou City and Infectious Disease Hospital of Tangshan City. All sites have established programs for the prevention of the transmission of HFMD. The study sites are arranged in ascending order of their initial letter, and assigned random site numbers using SAS random number generator. The treatment drug and identically matched placebo will be randomly assigned to A or B group with random drug number, and then the patients will be randomly assigned to A or B group after they are enrolled. Prior to the start of the study patients will undergo eligibility screening. All patients must sign the informed consent form prior to any screening procedures.

**Table 1** Cases Assignment in the 3 sites

| Sites | *Jinzhen* | Placebo | Site No. |
| --- | --- | --- | --- |
| Infectious Disease Hospital of Cangzhou City | 80 | 80 | 01 |
| The Fifth People’s Hospital of Guiyang City | 80 | 80 | 02 |
| Infectious Disease Hospital of Tangshan City | 70 | 70 | 03 |
| Total | 230 | 230 |  |

5.4 Blinding

This is a double-blind designed study with the investigator and patients under blinding. The protocol designer, investigators and statisticians will conduct their work in a relatively independent manner, and the statistical analysis will be conducted without unblinding.

The Randomization List will not be available at the study centre, to the monitors or project statisticians. If the identity of the test medication is necessary for patient management (in the case of a serious adverse event), it is necessary to ensure that adequate procedures are in place to ensure the integrity of the data.

A patient’s treatment assignment should only be unblinded when knowledge of the treatment is essential for the further management of the patient.

The principal investigator should make every attempt to contact the Sponsor before unblinding any patient’s treatment assignment, but must contact the Sponsor within one working day after the event and if appropriate, complete an adverse event form. In case of accidental unblinding, all the above procedures should be followed, although an adverse event form should not be completed.

**6. STUDY TREATMENT AND FOLLOW-UP**

6.1 Preparation Quality Control

The quality of *Jinzhen* oral liquid and its placebo, supplied by Kanion Pharmaceutical Co. Ltd, will be controlled by HPLC. The whole procedure of the quality control is in accordance with the standards of GMP and “Pharmacopoeia of the People's Republic of China in 2010” ^19^.

6.2 Treatment Regimens

Throughout the 7-day intervention period, the children should be kept in hospital.

6.2.1 Supportive care, according to "Diagnosis and treatment guidelines of HFMD (2010 edition)"(available at http://www.moh.gov.cn/publicfiles/business/htmlfiles/mohyzs/s3586/201004/46884.htm. Accessed on April 22^th^ 2010), is allowed in the study. The detail of the supportive care should be recorded in case report form (CRF).

6.2.2 Antipyretic care

6.2.2.1 Physical cooling method can be administered to children whose temperature is lower than 38.5℃;

6.2.2.2 Parents are also provided with ibuprofen suspension (Motrin, Johnson & Johnson) and instructed to administer it when their child’s temperature exceeds 38.5℃. The dosage of the ibuprofen suspension is shown in Table 2. The frequency of the use of ibuprofen suspension is depended on the normalization of temperature. Usually, it is administrated once every 4-6 hrs if the temperature does not decrease, but no more than 4 times a day.

**Table 2 The usage of the ibuprofen suspension in children aged 1-7 years**

| Age(year) | Weight( kg) | Dosage (ml) |
| --- | --- | --- |
| 1-3 | 10-15 | 4 |
| 4-6 | 16-21 | 5 |
| 7 | 22-27 | 8 |

6.2.2.3 If the child with HFMD are complicated by bacterial infection (WBC ≥10.0×10^9^/L, Neutrophils (%) ≥50%), the antibiotics will be taken if necessary. Cefuroxim injection is introduced to be intravenously used 3 or 4 times a day as totally 30-100mg per weight kilogram for 5 days. If the child is allergic to cefuroxim, azithromycin injection is also introduced to be intravenously used once a day as totally 8mg per weight kilogram for 5 days, or azithromycin granule is orally taken once a day as totally 12mg per weight kilogram (totally no more than 0.5g for one day).

6.2.3 Trial Regimen

The *Jinzhen* oral liquid and its placebo, supplied by Kanion Pharmaceutical Co. Ltd, will be administered three times a day for 7 days, followed by a 3-day follow-up. All liquid are sugar-free and supplied in licensed containers with child resistant caps. All parents are given a drug bottle containing either *Jinzhen* or placebo. Children will be randomly assigned to receive 1 ml of *Jinzhen* or placebo per kilogram body weight, and parents will be instructed to use a calibrated syringe to draw weight-dependent dosage from the drug bottle, and then administer the appropriate dose to their child with the needle tips being taken off. The amount of the every-day drug would be given for three times a day. Throughout the intervention period, parents will be asked to record their child’s temperature every 4 hrs under the help of specialized nurses. The first dose will be given under the supervision of the investigator and if possible, subsequent doses of *Jinzhen* or placebo should be administered at approximately the same time each day. The first 3 to 6 hrs after children are given the first dose and before any further drug is given^17^, is regarded as the “temperature efficacy period”. Since it's reported that the symptoms in some cases of HFMD disappeared after 48h under some supportive treatment, we asked parents to give the drugs three times a day in the first 2 days. And from Day 3 through Day 7, if their child’s symptoms persisted then, the parents should continue to give the drugs every 8 hrs until their child’s symptoms all disappear. At the end of Day 7, we discontinued the study drugs.

6.2.4 Disallowed Regimens

6.2.4.1 Do not use symptomatic treatment for oral ulcers and vesicles if not necessary.

6.2.4.2 Steroids are disallowed;

6.2.4.3 Do not use any Chinese herbal preparation with a same function of the trial drug.

6.2.4.4 Do not use any anti-viral therapy and interferon

6.2.4.5 Do not use antibiotics if not necessary [WBC＜10.0×10^9^/L, Neutrophils (%)＜50%]

6.3 Duration and Follow-up

6.3.1 Treatment Duration

This study of HFMD consists of a 3-7 day intervention and a 3-day follow-up. Investigator will determine the days of the intervention according to the heath status of the child. When the clinical signs and symptoms of the HFDM disappear, the treatment regimen will be discontinued. The withdrawal case due to poor compliance will be also continued to the follow-up procedure.

6.3.2 The Endpoint of Follow-up

The longest study duration is 10 day (7-day intervention and 3-day follow-up). All children will be required to stay in hospital for investigation. If any of the following conditions occurs, the follow-up will be discontinued:

6.3.2.1 Cure: the temperature returns to normal and the clinical signs and symptoms disappear;

6.3.2.2 Develop to severe cases: such as myocarditis, encephalitis, aseptic meningitis, pulmonary edema, and poliomyelitis-like paralysis;

6.3.2.3 Death: including the death caused by HFMD and other disease;

6.3.2.4 The follow-up procedure ends;

6.3.2.5 The parents or the guardian decline to continue the trial;

6.3.2.6 Loss to follow-up

6.3.3 The Manners of Follow-up

Each investigating sites will conduct follow-up in different manners, such as by telephone, outpatient service, home visiting or inpatient observation.

**7.** **OBSERVATION INFORMATION**

7.1 Baseline

7.1.1 Demographic parameters: including age, gender, height, weight, ethnics, residence, source of care.

7.1.2 Symptoms: including fever, cough, running nose, poor appetite, nausea, vomiting, salivating, diarrhea or constipation, discomfort, dysphoria, etc.

7.1.3 Signs: temperature, blood pressure, heart rate, respiratory rate, congestion of throat, oral ulcers, vesicles on hand or foot or buttock,

7.1.4 History: contact history with patients with HFMD, duration, coexisting illnesses, diagnosis patterns, history of drug allergy, treatment history;

7.1.5 Pathobiology test: Samples from the throat or stool will be sent to the laboratory of Chinese Centre for Disease Control and Prevention to test for virus and to find out which enterovirus caused the illness. The non-polio enteroviruses-infection status of children will be determined by means of non-polio enteroviruses (mainly as EV71 and Cox A16) RT-PCR testing.

7.1.6 Laboratory tests: three routine tests (blood, urine and stool), Chest X-ray, electrocardiogram (ECG), liver and kidney function test, myocardial zymogram, blood glucose for safety assessment

7.2 Observation Parameters

7.2.1 Temperature: Throughout the intervention period, parents will be asked to record their child’s armpit temperature every 4 hrs with the help of specialized nurses. The temperature test last 5 mins each time. If the temperature is tested normal (＜37℃) more than 3 times, the record of temperature can be stopped.

7.2.2 Symptoms: symptoms mentioned in section 7.1.2 are recorded every day.

7.2.3 Signs: signs mentioned in section 7.1.3 are recorded every day.

7.2.4 Pathobiology test: pathobiology test as mentioned in section 7.1.5 is tested when child is enrolled for diagnosis.

7.2.5 Laboratory tests:

7.2.5.1 Three routine tests (blood, urine and stool) and liver and kidney function test are taken for safety assessment before and after the study treatment;

7.2.5.2 Chest X-ray, ECG, myocardial zymogram, blood glucose is tested when the child is enrolled in the study. If the result is normal and the health status of the patients do not change to the worse (such as continuous high fever, discomfort of the heart, etc.), these test should not be taken after the child completes the study.

7.2.6 Frequency and dosage of ibuprofen: Parents are provided with ibuprofen suspension (Motrin, Johnson & Johnson) and instructed to administer it when their child’s temperature exceeds 38.5℃. The parents should record the time and the dosage of the use of ibuprofen.

**8. OUTCOME MEASURES**

8.1 Primary outcome measures

8.1.1 Time to the first disappearance of oral ulcers and vesicles on hand or foot within 10 days. We defined "oral ulcers and vesicles on hand or foot disappearing" as "no new ones appearing" according to the standard of this field. Moreover, the disappearance of oral ulcers and vesicles on hand or foot was determined by the investigators which were the experienced clinicians;

8.1.2 Time to the first normalization of temperature (fever clearance) including time to the first drop of temperature greater than 0.5℃ at 3, 6, 12 and 24 hrs after FIRST usage was taken. The rationale for time selection is based on a previous pharmacokinetic study on baicalin^27, 28^ which is the major active compound in *Jinzhen* oral liquid.

8.2 Secondary outcome measures

8.2.1 The proportion of children whose symptoms disappear within 10 days (assessed daily):

With the support from investigators, symptoms including discomfort, poor appetite, diarrhea, cough, dysphoria, etc. will be also collected and recorded in symptom diaries using a categorical scale by parents.

**8.2.2 Treatment failure rate (In previous edition of the protocol, this measure was** **the incidence of severe case. However, for overall assessment, this edition changes it to the treatment failure rate)**

Treatment failure rate is another important outcome measure for efficacy evaluation, which is defined as the proportion of patients whose infection has not been controlled during the 7-day intervention and 3-day follow-up period. Based on a previous study^21^, treatment failure is defined as the occurrence of any of the following conditions: 1) severe disorders (such as encephalitis); or 2) WBC counts＞10,000/ mm^3^.

8.2.3 The frequency and dose of ibuprofen (if used)

8.2.4 Safety assessment

Throughout the entire intervention period, we will monitor all adverse events, using a standard adverse-event case report form at each visit. This form includes a description of all unanticipated events and undesirable experiences, particularly exacerbations of HFMD symptoms.

**8.2.5 Direct economic cost (removed in the final edition with an approval from IRB)**

**9 SAFETY INSTRUCTIONS AND GUIDANCE**

9.1 Previous safety information

According to phase Ⅱ and Ⅲ of the clinical trials and the decade-use in clinical practice, no serious adverse events have been reported, which indicates that it may be a safe drug for children.

9.2 Adverse Events (AEs) and Laboratory Abnormalities

9.2.1 Clinical AEs

Based on the International Conference of Harmonisation [ICH], an AE is any untoward medical occurrence in a patient or clinical investigation subject administered a pharmaceutical product and which does not necessarily have a causal relationship with this treatment. An AE can therefore be any unfavorable and unintended sign [including an abnormal laboratory finding], symptom, or disease temporally associated with the use of a medicinal [investigational] product, whether or not considered related to the medicinal [investigational] product. Pre-existing conditions which worsen during a study are to be reported as AEs.

For all adverse events, the following must be assessed and recorded on the adverse events form of the CRF: intensity, relationship to study drug, action taken regarding study drug, and outcome to date.

9.2.1.1 Intensity

All clinical AEs encountered during the clinical study will be reported on the AE Form of the CRF. In addition, all AEs will be captured during the open label treatment period and the period of safety follow up.

Intensity of AEs will be graded using the following criteria:

Mild: discomfort noticed but no disruption of normal daily activity.

Moderate: discomfort sufficient to reduce or affect daily activity.

Severe: inability to work or perform normal daily activity

Life Threatening: represents an immediate threat to life

Relationship of the AE to the treatment should always be assessed by the investigator. Description of scales can be found in Appendix 2).

9.2.1.2 Serious Adverse Events [Immediately Reportable to Sponsor]

A Serious Adverse Event (SAE) is any experience that suggests a significant hazard, contraindication, side effect or precaution. It is any AE that at any dose fulfils at least one of the following criteria:

– is fatal; [results in death; NOTE: death is an outcome, not an event]

– is Life-Threatening [NOTE: the term “Life-Threatening” refers to an event in which the subject was at immediate risk of death at the time of the event; it does not refer to an event which could hypothetically have caused a death had it been more severe].

– required in-patient hospitalization or prolongation of existing hospitalization;

– results in persistent or significant disability/incapacity;

– is a congenital anomaly/birth defect;

– is medically significant or requires intervention to prevent one or other of the outcomes listed above.

The full requirements of the ICH Guideline for Clinical Safety Data Management, Definitions and Standards for Expedited Reporting, Topic E2 will be adhered to [see Appendix 3].

9.2.2 Treatment and Follow-up of AEs

AEs, especially those for which the relationship to test drug is not “unrelated”, should be followed up until they have returned to baseline status or stabilized. If a clear explanation is established, it should be recorded on the CRF.

9.2.3 Laboratory Test Abnormalities

Laboratory test results will appear on electronically produced laboratory reports submitted directly from the central laboratory.

Any laboratory result abnormality fulfilling the criteria for a SAE should be reported as such, in addition to being recorded as an AE in the CRF.

Any treatment-emergent abnormal laboratory result which is clinically significant, i.e., meeting one or more of the following conditions, should be recorded as a single diagnosis on the AE Form in the CRF:

 Accompanied by clinical symptoms

 Leading to a change in study medication [e.g., interruption or permanent discontinuation]

Requiring a change in concomitant therapy [e.g. addition of, interruption of, discontinuation of, or any other change in a concomitant medication, therapy or treatment].

9.2.4 Follow-up of Abnormal Laboratory Test Values

In the event of clinically significant unexplained abnormal laboratory test values, the tests should be repeated and followed up until they have returned to the normal range and/or an adequate explanation of the abnormality is found. If a clear explanation is established it should be recorded on the CRF.

9.3 Handling of Safety Parameters

9.3.1 Reporting of AEs

The following AE types are considered exempt from the above, and should not be recorded in the CRF: Flares of Rheumatoid Arthritis or worsening of the signs and symptoms of rheumatoid arthritis. These events will be captured as specific outcomes on relevant pages of the CRF.

9.3.2 Reporting of Serious Adverse Events [Immediately Reportable]

Any clinical AE or abnormal laboratory test value that is *serious* [as defined in Section 9.2.1.2 above] and which occurs during the course of the study, regardless of the treatment arm, must be reported to the Sponsor within *one* working day (24 h) of the investigator becoming aware of the event [expedited reporting].

The site is required to complete a paper SAE form provided by the Sponsor. The completed SAE page(s) and corresponding fax cover sheet should be faxed immediately upon completion to 86-10-64014411-2805.

Relevant follow up information should be submitted as soon as it becomes available.

Fatal or life-threatening events considered to be caused by *Jinzhen* must be telephoned to the Protocol Co Chairs immediately (Prof. Zhong Wang: 86-10-64014411-3308 or 86-0-13601315446).

The following SAEs occurring after the patient signs the informed consent form (ICF) and before investigational product dosing will be reported to the Sponsor: a) SAEs related to trial procedures; b) all the SAEs related to concomitant medications. AEs should be recorded according to local requirements.

For all patients, serious adverse events occurring during study treatment periods or which come to the attention of the investigator during the Safety follow-up visits must be reported, whether considered treatment-related or not. In addition, a serious adverse event that occurs after this time, if considered related to test drug, should also be reported.

A death occurring during the study or which comes to the attention of the investigator during the Safety follow-up visits must be reported, whether considered treatment-related or not; deaths will be considered unexpected in this trial and subject to emergency reporting rules (see above).

Investigators are required to promptly notify their respective IRB of all adverse drug reactions that are both serious and unexpected. This generally refers to SAEs that are not already identified in the Investigator Brochure and that are considered by the investigator to be possibly or probably related to study drug.

Investigators must immediately forward to their IRB any written safety report or update provided by the Sponsor (e.g., Investigator Brochure Update, safety amendments and updates, etc.)

The definition and reporting requirements of ICH Guideline for Clinical Safety Data Management, Definitions and Standards for Expedited Reporting, Topic E2 will be adhered to. Complete information can be found in Appendix **5**

**10. DATA COLLECTION, MANAGEMENT AND QUALITY ASSURANCE**

10.1 Collection and Fulfillment of CRF

The investigators fill in the 3-copy CRF of every patient enrolled. After the monitors review the completed CRF, the first copy of the CRF will be sent to the statisticians for data collection and management. Once the first copy of the CRF is submitted, the CRF will not be done any modification.

10.2 Data Collection and management

The statisticians are responsible for data Collection and management. Data management will be performed via EpiData 3.0. To assure the accuracy of the data, two statisticians should record the data from the CRF independently. Once there are any queries about the CRF, the statisticians should fill in a data queries form (DQF), and send it to the monitors. Then the monitors require the investigators to resolve the queries as soon as possible. The statisticians will do the data modification according to the correspondence from the investigators. The statisticians could send another DQF if necessary.

10.3 Data Lock

After confirming the accuracy of the database established from the CRF, the investigators, sponsor, statisticians will agree to lock the data. The locked database should not be done any modification. Any problems found after the data lock will be resolved in the procedure of statistical analysis.

**11. STASTISTICAL** **CONSIDERATIONS AND ANALYTICAL PLAN**

11.1 Sample Size Calculation

Since the main study goal is to prove the efficacy of *Jinzhen* oral liquid, we will use the log-rank test to compare the time to the first normalization of body temperature between treatment group and control. Suppose the survivor function of the time to the first normalization of body temperature is S_1_(x) in the control group, and S_2_(x) in the treatment group, the log-rank method test the following null hypothesis:

against the two-side alternative of the two survivor functions are not all equal. The SAS LIFTTEST procedure will be used. For the two-sided log-rank test^29-31^, there is no explicit formulae for sample size calculation, we used SAS PROC POWER to calculate the sample size needed. We make a balanced design; the same number of subjects is allocated to each group.

From former studies^32^, we know that the average time to the first normalization of temperature for HFMD patient is 2.68 days. The pilot study of *Jinzhen* oral liquid for HFMD (unpublished) showed that it could reduce the time of fever clearance to 1.05 day and reduce the time to the first disappearance of oral ulcers and vesicles from 5.46 day to 4.05 day. Therefore, we estimate using of *Jinzhen* oral liquid can speedup the time of fever clearance for HFMD patient by 16-24 hours and the time to the first disappearance of oral ulcers and vesicles by 1-1.4 days. We assume exponential distribution for the event times in the two groups in sample size calculation. We choose significance level 0.025 according to α adjusted by Bonferroni method, target test power 0.80. The following SAS code produced a total sample size of 422. We added 20% more children to allow for loss to follow up. Therefore, the sample size is enlarged to 460, with 230 subjects in each group.

11.2 Analysis Sets

11.2.1Full analysis set

The full analysis set (FAS) is used to describe the study populations included in the efficacy and safety analyses. The “full analysis set” includes all patients randomized to treatment who received at least one dose of the assigned treatment. This term is as close as possible to the intention-to-treat (ITT) ideal of including all randomized subjects.

11.2.2 Per Protocol Set

The per protocol set (PPS)of subjects, sometimes described as the "valid cases", the "efficacy" sample or the "evaluable subjects" sample, defines a subset of the subjects in the full analysis set who are more compliant with the protocol and is characterised by criteria such as the following:

1.the completion of a certain pre-specified minimal exposure to the treatment regimen; 2.the availability of measurements of the primary variable(s);

3.the absence of any major protocol violations including the violation of entry criteria.

11.2.3 Safety Analysis Set

The safety analysis set (SS) is defined as a subset of the randomized analysis set that includes subjects who took at least 1 dose of investigational product and provided at least one assessment of safety.

11.3 Statistical Methods

11.3.1 Statistical significant level

All *P* values were two-side. Statistical significant level was set to be 0.05.

11.3.2 Analysis Populations

Define the 3 analysis data sets (FAS, PPS, SS) via confirming the enrolled cases and completed cases in the trial. Patients who prematurely withdraw or were rejected from the study should be listed the reasons. The baseline analysis will be performed in FAS. All efficacy analyses and safety analyses will be performed on an ITT basis. Besides, the efficacy analyses will also be performed in PPS in order to enhance the reliability of the result if we get the similar result in both PPS and FAS. However, if the results from FAS are not in accordance with that from the PPS, the results from FAS will be considered as the main outcome.

11.3.3 Baseline Analysis

Demographic variables and symptom variables related to HFMD at baseline were described and compared between treated and control groups to show the homogeneity. Student’s t test or analysis of variance (ANOVA) was applied for quantitative data. Chi-square test or Fisher’s exact method was adopted for qualitative data. Wilcoxon rank-sum test was conducted for ranked data.

11.3.4 Efficacy Analysis

The primary outcome measures are time-to-event variables with survival curves made by the Kaplan-Meier method, and compared between the two groups via log-rank test stratified respectively by age, course of HFMD, medical history, contact history and so on. Cochran-Mantel-Haenszel *P* values are calculated. Cox propotional risk model is used to estimate the hazard ratios (HRs) and 95% confidence intervals (CIs) with covariates adjustment including age, course of HFMD, medical history, contact history with the patient with HFMD and so on.

The proportion of children whose symptoms disappear within 10 days (assessed daily), one of the secondary outcome measures, is compared between the two groups by Fisher’s exact method and binomial 95% CIs. If used, the frequency and dose of ibuprofen are compared by Wilcoxon rank-sum test. **The incidence of severe case (changed to treatment failure rate in the last edition)** is also compared by Fisher’s exact method.

11.3.5 Safety Analysis

Descriptive statistics was used for medical adverse events (AE). Incidence rate of AE were compared by Fisher’s exact method. Experimental variables were compared before and after the therapy through paired-t test, and also contrasted between groups through Student’s t test.

11.4 Other Statistical Considerations

Data entry was done by EpiData 3.0 software. Quality control(QC) including consistency check and blind logic check were done. QC and statistical analysis were performed by Statistical Analysis System (SAS) version 9.1.3 (SAS institute, Cary, NC).

**12. ETHICAL ASPECTS**

12.1 Local Regulations/Declaration of Helsinki

The investigator will ensure that this study is conducted in full conformance with the principles of the “Declaration of Helsinki” or with the laws and regulations of the country in which the research is conducted, whichever affords the greater protection to the patient. The study must fully adhere to the principles outlined in “Guideline for Good Clinical Practice (GCP)” ICH Tripartite Guideline [January 1997] or with local law if it affords greater protection to the patient. to the patient. For studies conducted in China, the investigator will ensure compliance with GCP issued by SFDA in China.

12.2 Informed Consent

It is the responsibility of the investigator, or a person designated by the investigator [if acceptable by local regulations], to obtain written informed consent from each guardian of the child participating in this study after adequate explanation of the aims, methods, objectives and potential hazards of the study. It must also be explained to the patients that they are completely free to refuse to enter the study or to withdraw from it at any time for any reason. Appropriate forms for obtaining written informed consent for the protocol and optional blood sample collection will be provided by the sponsor.

In the case where the child’s guardian is unable to read or write, an impartial witness should be present during the entire informed consent discussion and every subsequent visit. After the guardian has orally consented to participation in the trial, the witness’ signature on the form will attest that the information in the consent form was accurately explained and understood. The CRFs for this study contain a section for documenting informed guardian consent, and this must be completed appropriately. If new safety information results in significant changes in the risk/benefit assessment, the consent form should be reviewed and updated if necessary. All guardians [including whose child already being treated] should be informed of the new information, given a copy of the revised form and give their consent to continue in the study.

12.3 Institutional Review Board (IRB)

This protocol and any accompanying material provided to the subject [such as patient information sheets or descriptions of the study used to obtain informed consent], will be submitted by the investigator to the IRB of the Institute of Basic Clinical Research, China Academy of Chinese Medical Sciences. Approval from the board must be obtained before starting the study, and should be documented in a letter to the investigator specifying the date on which the board met and granted the approval.

Any modifications made to the protocol after receipt of the IRB approval must also be submitted by the investigator to the board in accordance with local procedures and regulatory requirements.

**13. CONDITIONS FOR MODIFYING THE PROTOCOL**

Protocol modifications to ongoing studies must be made only after consultation between an appropriate representative of the sponsor and the investigator representatives. Protocol modifications must be prepared by a representative of the Sponsor and initially reviewed and approved by the Protocol Co Chairs and Biostatistician.

All protocol modifications must be submitted to the IRB for information and approval in accordance with local requirements, and to Regulatory Agencies if required. Approval must be awaited before any changes can be implemented, except for changes necessary to eliminate an immediate hazard to trial patients, or when the change[s] involves only logistical or administrative aspects of the trial [e.g. change in monitor[s], change of telephone number[s].

**14. CONDITIONS FOR TERMINATING THE STUDY**

Both the sponsor and the investigator reserve the right to terminate the study at any time. Should this be necessary, both parties will arrange the procedures on an individual study basis after review and consultation. In terminating the study, the Sponsor and the investigator will assure that adequate consideration is given to the protection of the patient’s interests.

**15. STUDY DOCUMENTATION, CRFS AND RECORD KEEPING**

15.1 Investigator's Files / Retention of Documents

The Investigator must maintain adequate and accurate records to enable the conduct of the study to be fully documented and the study data to be subsequently verified. These documents should be classified into two different separate categories: investigator's study file, and patient clinical source documents.

The Investigator’s Study File will contain the protocol/amendments, a paper representation of the CRF, Institutional Review Board and governmental approval with correspondence, sample informed consent, drug records, staff curriculum vitae and authorization forms and other appropriate documents/correspondence etc. In addition at the end of the study the investigator will receive the patient data, which includes an audit trail containing a complete record of all changes to data, query resolution correspondence and reasons for changes, in human readable format on compact disk which also has to be kept with the Investigator’s Study File.

Patient clinical source documents [usually defined by the project in advance to record key efficacy/safety parameters independent of the CRFs] would include patient hospital/clinic records, physician’s and nurse’s notes, appointment book, original laboratory reports, ECG, X-rays, pathology and special assessment reports, signed informed consent forms, consultant letters, and subject screening and enrollment logs.

In all sites the Investigator must keep these two categories of documents (including the archival compact disk) on file for at least 10 years after completion or discontinuation of the study. After that period of time the documents may be destroyed once the site has provided written notification 60 days prior to destruction of the documents, subject to local regulations. No records should be disposed of without the written approval of the Sponsor.

Should the Investigator wish to assign the study records to another party or move them to another location, the Sponsor must be notified in advance.

If the Investigator can not guarantee this archiving requirement at the investigational site for any or all of the documents, special arrangements must be made between the Investigator and the Sponsor to store these in a sealed container[s] outside of the site so that they can be returned sealed to the Investigator in case of a regulatory audit. Where source documents are required for the continued care of the patient, appropriate copies should be made for storing outside of the site.

15.2 Source Documents and Background Data

Study monitors will perform ongoing source data verification (SDV) to confirm that critical protocol data (i.e., source data) entered on the CRFs by authorized site personnel are accurate, complete, and verifiable from source documents. In no case is the CRF to be considered as source data for this trial.

Source documents are where subject data are recorded and documented for the first time. They include, but are not limited to hospital records, clinical and office charts, laboratory notes, memoranda, subject diaries or evaluation checklists, pharmacy dispensing records, recorded data from automated instruments, copies of transcriptions that are certified after verification as being accurate and complete, microfiche, photographic negatives, microfilm or magnetic media, X-rays, subject files, and records kept at the pharmacy, laboratories, and medico-technical departments involved in a clinical trial.

The investigator shall supply the sponsor on request with any required background data from the study documentation or clinic records. This is particularly important when errors in data transcription are suspected. In case of special problems and/or governmental queries or requests for audit inspections, it is also necessary to have access to the complete study records, provided that patient confidentiality is protected.

Source documents that are required to verify the validity and completeness of data transcribed on the CRFs must never be obliterated or destroyed.

To facilitate SDV, the investigator(s) and institutions must provide the Sponsor direct access to applicable source documents and reports for trial-related monitoring, Sponsor audits, and IRB review. The investigational site must also allow inspection by applicable regulatory authorities.

15.3 Audits and Inspections

The investigator should understand that source documents for this trial should be made available to appropriately qualified personnel from the Sponsor’s Quality Assurance group or its designers or to health authority inspectors after appropriate notification. The verification of the CRF data must be by direct inspection of source documents.

15.4 Case Report Forms

Data for this study will be managed on computer via Epidata 3.0 from Case Report Forms. The data is entered on to the computer using the off-line mode. An audit trail will maintain a record of initial entries and changes made; reasons for change; time and date of entry; and user name of person authorizing entry or change. The investigator will connect on a regular basis, using an analog phone line, and the data will be transferred directly to the Sponsor’s database.

For each patient randomized, a CRF must be completed and signed by the principal investigator or authorized delegate from the study staff. This also applies to records for those patients who fail to complete the study [even during a pre-randomization screening period if a CRF was initiated]. If a patient withdraws from the study, the reason must be noted on the CRF. If a patient is withdrawn from the study because of a treatment-limiting AE, thorough efforts should be made to clearly document the outcome.

The investigator should ensure the accuracy, completeness, and timeliness of the data reported to the sponsor in the CRFs and in all required reports.

**16. MONITORING THE STUDY**

It is understood that the Sponsor’s monitor will contact and visit the investigator regularly and will be allowed, on request, to inspect the various records of the trial [CRFs and other pertinent data] provided that patient confidentiality is maintained in accord with local requirements. Site visits will be conducted by an authorized Sponsor representative to inspect study data, subjects’ medical records and CRFs in accordance with current ICH GCP and GCP of SFDA in China.

It will be the monitor's responsibility to inspect the CRFs at regular intervals throughout the study, to verify the adherence to the protocol and the completeness, consistency and accuracy of the data being entered on them. The monitor should have access to laboratory test reports and other patient records needed to verify the entries on the CRF. The investigator [or his/her deputy] agrees to cooperate with the monitor to ensure that any problems detected in the course of these monitoring visits are resolved.

**17. CONFIDENTIALITY OF TRIAL DOCUMENTS AND SUBJECT RECORDS**

The investigator must assure that patients’ anonymity will be maintained and that their identities are protected from unauthorized parties. On CRFs or other documents submitted to the sponsor, subjects should not be identified by their names, but by an identification code. The investigator should keep a subject enrollment log showing codes, names and addresses. The investigator should maintain documents not for submission to the Sponsor, e.g., patients’ written consent forms, in strict confidence.

**18. PUBLICATION OF DATA AND PROTECTION OF TRADE SECRETS**

The results of this study may be published or presented at scientific meetings. If this is foreseen, the investigator agrees to submit all manuscripts or abstracts to the Sponsor prior to submission. This allows the sponsor to protect proprietary information and to provide comments based on information from other studies that may not yet be available to the investigator.

In accord with standard editorial and ethical practice, the Sponsor will generally support publication of multicenter trials only in their entirety and not as individual center data. In this case, a coordinating investigator will be designated by mutual agreement.

Any formal publication of the study in which input of the Sponsor personnel exceeded that of conventional monitoring will be considered as a joint publication by the investigator and the appropriate the Sponsor personnel. Authorship will be determined by mutual agreement.

**19. REFERENCES**

1.Lum LC, Wong KT, Lam SK , et al. Fatal enterovirus 71 encephalomyelitis. J Pediatr 1998;133(6):795-8.

2.Li LJ. Review of hand, foot and mouth disease. Frontiers of Medicine in China 2010;4(2):139-146

3.Ma E, Lam T, Chan KC, Wong C, Chuang SK. Changing epidemiology of hand, foot, and mouth disease in Hong Kong, 2001-2009. Jpn J Infect Dis 2010;63:422-6.

4. Ho M, Chen ER, Hsu KH, et al. An epidemic of enterovirus 71 infection in Taiwan. N Engl J Med 1999;341:929-35.

5.Chang LY, Lin TY, Hsu KH, et al. Clinical features and risk factors of pulmonary oedema after enterovirus-71-related hand, foot, and mouth disease. Lancet 1999;354:1682-6.

6. Chan KP, Goh KT, Chong CY, Teo ES, Lau G, Ling AE. Epidemic hand, foot and mouth disease caused by human enterovirus 71, Singapore. Emerg Infect Dis 2003;9:78-85.

**7**.Fujimoto T, Chikahira M, Yoshida S,et al. Outbreak of central nervous system disease associated with hand, foot, and mouth disease in Japan during the summer of 2000: detection and molecular epidemiology of enterovirus 71. Microbiol Immunol 2002;46:621-7.

8. Ministry of Health reports the information on prevention and control work of HFMD. Beijing: Information Office of Ministry of Health of the People’s Republic of China, 2010 (Accessed June 24, 2010, at http://www.moh.gov.cn/publicfiles/business/htmlfiles/mohbgt/ s3582/201006/47871.htm)

9.Toida M, Watanabe F, Goto K, Shibata T. Usefulness of low-level laser for control of painful stomatitis in patients with hand-foot-and-mouth disease. J Clin Laser Med Surg 2003;21:363-7.

10.Wang SM, Lei HY, Huang MC, et al. Therapeutic efficacy of milrinone in the management of enterovirus 71-induced pulmonary edema. Pediatr Pulmonol 2005;39:219-23.

11. Lau TF, Leung PC, Wong EL,et al. Using herbal medicine as a means of prevention experience during the SARS crisis. Am J Chin Med 2005;33:345-56.

12. Wang YT, Chan CH, Su ZY, Chen CL. Homology modeling, docking, and molecular dynamics reveal HR1039 as a potent inhibitor of 2009 A(H1N1) influenza neuraminidase. Biophys Chem 2010;147:74-80.

13.Wang H, Li W, Li J, et al. The aqueous extract of a popular herbal nutrient supplement, Angelica sinensis, protects mice against lethal endotoxemia and sepsis. J Nutr 2006;136:360-5.

14. Ashafa AO, Yakubu MT, Grierson DS, Afolayan AJ. Evaluation of aqueous extract of Felicia muricata leaves for anti-inflammatory, antinociceptive, and antipyretic activities. Pharm Biol 2010;48:994-1001.

15. Kupeli E, Tosun A, Yesilada E. Assessment of anti-inflammatory and antinociceptive activities of Daphne pontica L. (Thymelaeaceae).J Ethnopharmacol 2007;113:332-7.

16. Ma SC, Du J, But PP, et al. Antiviral Chinese medicinal herbs against respiratory syncytial virus. J Ethnopharmacol 2002; 79:205-11.

17. Hirschfeld G, Weber L, Renkl A, Scharffetter-Kochanek K, Weiss JM. Anaphylaxis after Oseltamivir (Tamiflu) therapy in a patient with sensitization to star anise and celery-carrot-mugwort-spice syndrome. Allergy 2008;63:243-4.

18. Lin TY, Liu YC, Jheng JR, et al. Anti-enterovirus 71 activity screening of chinese herbs with anti-infection and inflammation activities. Am J Chin Med 2009;37:143-58.

19. Pharmacopoeia Commission of the People's Republic of China. Pharmacopoeia of the People's Republic of China (Chinese Edition 2010) Vol.Ⅰ. Beijing, China: China Medical Science Press, 2010: 829-30

20. Tsai CC, Lin MT, Wang JJ, Liao JF, Huang WT. The antipyretic effects of baicalin in lipopolysaccharide-evoked fever in rabbits. Neuropharmacology. 2006;51:709-17..

21. Liu L, Yuan S, Long Y, et al. Immunomodulation of Rheum tanguticum polysaccharide (RTP) on the immunosuppressive effects of dexamethasone (DEX) on the treatment of colitis in rats induced by 2,4,6-trinitrobenzene sulfonic acid. Int Immunopharmacol 2009;9:1568-77.

22. Hsieh CJ, Hall K, Ha T, Li C, Krishnaswamy G, Chi DS. Baicalein inhibits IL-1beta- and TNF-alpha-induced inflammatory cytokine production from human mast cells via regulation of the NF-kappaB pathway. Clin Mol Allergy 2007;5:5.

23. Woo KJ, Lim JH, Suh SI, et al. Differential inhibitory effects of baicalein and baicalin on LPS-induced cyclooxygenase-2 expression through inhibition of C/EBPbeta DNA-binding activity. Immunobiology 2006;211:359-68.

24.Yoon SB, Lee YJ, Park SK, et al. Anti-inflammatory effects of Scutellaria baicalensis water extract on LPS-activated RAW 264.7 macrophages. J Ethnopharmacol 2009;125:286-90.

25. Kim JK, Oh SM, Kwon HS, Oh YS, Lim SS, Shin HK. Anti-inflammatory effect of roasted licorice extracts on lipopolysaccharide-induced inflammatory responses in murine macrophages. Biochem Biophys Res Commun 2006;345:1215-23.

26.Utsunomiya T, Kobayashi M, Pollard RB, Suzuki F. Glycyrrhizin, an active component of licorice roots, reduces morbidity and mortality of mice infected with lethal doses of influenza virus. Antimicrob Agents Chemother 1997;41:551-6.

27. Xiao L, Wang F, Li HD, Zhao XY.Pharmacokinetic study on baicalin of Qingkailing injection in rats. Zhongguo Zhong Yao Za Zhi 2007;32:2534-8.

28. Muto R, Motozuka T, Nakano M, Tatsumi Y, Sakamoto F, Kosaka N. The chemical structure of new substance as the metabolite of baicalin and time profiles for the plasma concentration after oral administration of sho-saiko-to in human. Yakugaku Zasshi 1998;118:79-87.

29. Mantel N. Evaluation of survival data and two new rank order statistics arising in its consideration. Cancer Chemother Rep 1966;50:163–70.

30. Peto R, Peto J. Asymptotically Efficient Rank Invariant Test Procedures. Journal of the Royal Statistical Society Series A (General) 1972;135: 185–207.

31.Schoenfeld D. The asymptotic properties of nonparametric tests for comparing survival distributions. Biometrika 1981;68: 316–19.

32.Gao SY. A 56-case clinical study on Reduling injection for hand, foot, and mouth disease. China Medical Herald 2009;22:244-5

**APPENDICES**

**Appendix 1 Fingerprint electropherogram and chemical structures of the *Jinzhen* oral liquid**

(1) Fingerprint electropherogram of *Jinzhen* oral liquid (a) and placebo(b)


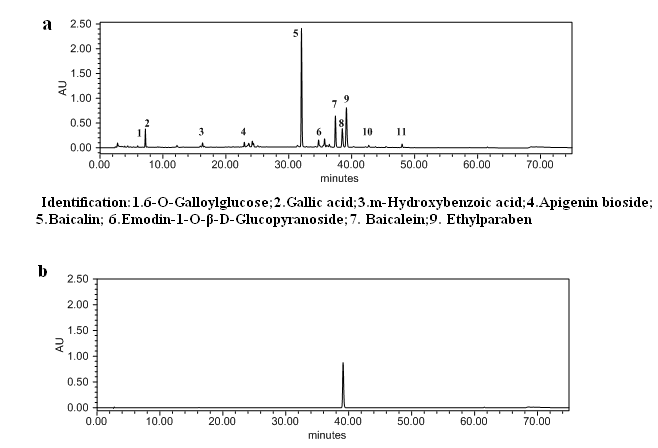


(2) Chemical structures of the major active compounds of *Jinzhen* oral liquid


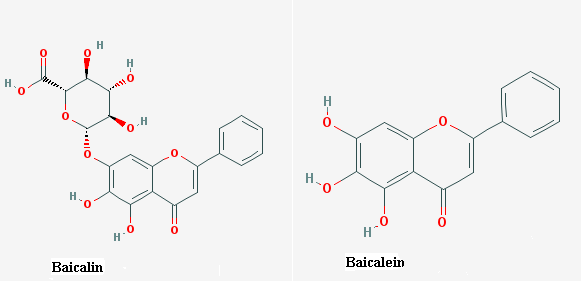


**Appendix 2. Adverse Events Categories for Determining Relationship to Test Drug**

**PROBABLE** [must have first three]

This category applies to those AEs which are considered, with a high degree of certainty, to be related to the test drug. An AE may be considered probable, if:

1. It follows a reasonable temporal sequence from administration of the drug.

2. It cannot be reasonably explained by the known characteristics of the subject’s clinical state, environmental or toxic factors, or other modes of therapy administered to the subject.

3. It disappears or decreases on cessation or reduction in dose. [There are important exceptions when an AE does not disappear upon discontinuation of the drug, yet drug-relatedness clearly exists; e.g., [1] bone marrow depression, [2] tardive dyskinesias.]

4. It follows a known pattern of response to the suspected drug.

5. It reappears upon rechallenge.

**POSSIBLE** [must have first two]

This category applies to those AEs in which the connection with the test drug administration appears unlikely but cannot be ruled out with certainty. An AE may be considered possible if, or when:

1. It follows a reasonable temporal sequence from administration of the drug.

2. It may have been produced by the subject’s clinical state, environmental or toxic factors, or other modes of therapy administered to the subject.

3. It follows a known pattern of response to the suspected drug.

**REMOTE** [must have first two]

In general, this category is applicable to an AE which meets the following criteria:

1. It does not follow a reasonable temporal sequence from administration of the drug.

2. It may readily have been produced by the subject’s clinical state, environmental or toxic factors, or other modes of therapy administered to the subject.

3. It does not follow a known pattern of response to the suspected drug.

4. It does not reappear or worsen when the drug is readministered.

**UNRELATED**

This category is applicable to those adverse events which are judged to be clearly and incontrovertibly due only to extraneous causes (disease, environment, etc.) and do not meet the criteria for drug relationship listed under remote, possible, or probable.


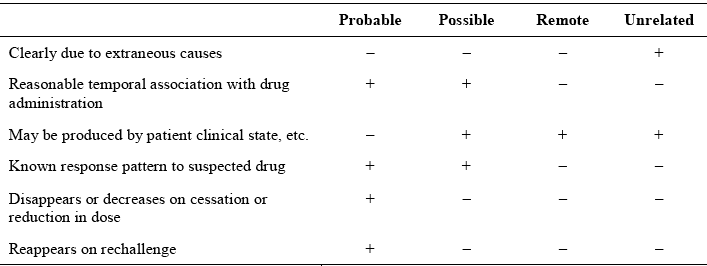


**Appendix 3. ICH Guidelines for Clinical Safety Data Management, Definitions and Standards for Expedited Reporting, Topic E2**

A serious adverse event is any experience that suggests a significant hazard, contraindication, side effect or precaution. It is any AE that at any dose fulfills at least one of the following criteria:

– is fatal; [results in death] [NOTE: death is an outcome, not an event]

– is Life-Threatening [NOTE: the term “Life-Threatening” refers to an event in which the patient was at immediate risk of death at the time of the event; it does not refer to an event which could hypothetically have caused a death had it been more severe].

– required in-patient hospitalization or prolongation of existing hospitalization;

– results in persistent or significant disability/incapacity;

– is a congenital anomaly/birth defect;

– is medically significant or requires intervention to prevent one or other of the outcomes listed above

Medical and scientific judgment should be exercised in deciding whether expedited reporting to the sponsor is appropriate in other situations, such as important medical events that may not be immediately life-threatening or result in death or hospitalization but may jeopardize the patient or may require intervention to prevent one of the outcomes listed in the definitions above. These situations should also usually be considered serious.

Examples of such events are intensive treatment in an emergency room or at home for allergic bronchospasm; blood dyscrasias or convulsions that do not result in hospitalization; or development of drug dependency or drug abuse.

An unexpected AE is one, the nature or severity of which is not consistent with the applicable product information.

Causality is initially assessed by the investigator. For Serious Adverse Events, possible causes of the event is indicated by selecting one or more options. (Check all that apply)

– Pre-existing/Underlying disease - specify

– Study treatment – specify the drug(s) related to the event

– Other treatment (concomitant or previous) – specify

– Protocol-related procedure

– Other (e.g. accident, new or intercurrent illness) – specify

The term severe is a measure of intensity, thus a severe AE is not necessarily serious. For example, nausea of several hours' duration may be rated as severe, but may not be clinically serious.

Any serious adverse event occurring during the study or which comes to the attention of the investigator within 15 days after stopping the treatment or during the protocol-defined follow-up period, if this is longer, whether considered treatment-related or not, must be reported. In addition, any serious adverse event that occurs after this time, if considered related to test “drug”, should be reported.

Such preliminary reports will be followed by detailed descriptions later which will include copies of hospital case reports, autopsy reports and other documents when requested and applicable.

For serious adverse events, the following must be assessed and recorded on the AEs page of the CRF: intensity, relationship to test substance, action taken, and outcome to date.

The investigator must notify the Ethics Review Committee/Institutional Review Board of a serious adverse event in writing as soon as is practical and in accordance with international and local laws and regulations.
