# Supplementary material for: Therapeutic Effect of Jinzhen Oral Liquid for Hand Foot and Mouth Disease: A Randomized, Multi-Center, Double-Blind, Placebo-Controlled Trial
Source: PLoS One. 2014 Apr 10;9(4):e94466. doi: 10.1371/journal.pone.0094466 (PMC3983189; doi:10.1371/journal.pone.0094466)
Supplement: Table S1 — The major components in Jinzhen oral liquid. (DOC) [file pone.0094466.s005.doc]

**Table S1. The major components in *Jinzhen* oral liquid**

| **Name in Chinese** | **Name in English** | **Name in Latin** |
| --- | --- | --- |
| **平贝母（***Pingbeimu***）** | ussuri fritillary bulb | *fritillaria ussuriensis maxim* |
| **大黄（***Dahuang***）** | rhubarb | *Radix et Rhizoma Rhei* |
| **黄芩（***Huangqin***）** | baikal skullcap root | *Radix Scutellariae* |
| **青礞石（***Qingmengshi***）** | chlorite schist | *Lapis Chloriti* |
| **生石膏（***Shengshigao***）** | gypsum | *Gypsum Fibrosum* |
| **人工牛黄（***Rengong Niuhuang***）** | artificial bezoar | *Calculus Bovis Artifactus* |
| **甘草（***Gancao***）** | Liquorice Root | *Radix Glycyrrhizae* |
